# Defects-Rich Heterostructures Trigger Strong Polarization Coupling in Sulfides/Carbon Composites with Robust Electromagnetic Wave Absorption

**DOI:** 10.1007/s40820-024-01515-0

**Published:** 2024-09-27

**Authors:** Jiaolong Liu, Siyu Zhang, Dan Qu, Xuejiao Zhou, Moxuan Yin, Chenxuan Wang, Xuelin Zhang, Sichen Li, Peijun Zhang, Yuqi Zhou, Kai Tao, Mengyang Li, Bing Wei, Hongjing Wu

**Affiliations:** 1 School of Physics, Xidian University, Xi’an, 710071 People’s Republic of China; 2 School of Advanced Materials and Nanotechnology, State Key Discipline Laboratory of Wide Band Gap Semiconductor Technology, Xidian University, Xi’an, 710071 People’s Republic of China; 3 School of Microelectronics, Xidian University, Xi’an, 710071 People’s Republic of China; 4 School of Telecommunication Engineering, Xidian University, Xi’an, 710071 People’s Republic of China; 5 MOE Key Laboratory of Material Physics and Chemistry Under Extraordinary, School of Physical Science and Technology, Northwestern Polytechnical University, Xi’an, 710072 People’s Republic of China; 6 The Ministry of Education Key Laboratory of Micro and Nano Systems for Aerospace, School of Mechanical Engineering, Northwestern Polytechnical University, Xi’an, 710072 People’s Republic of China

**Keywords:** Defects-rich heterointerfaces; Sulfides; Polarization coupling; Electromagnetic wave absorption

## Abstract

Defects-rich heterointerfaces integrated with adjustable crystalline phases and atom vacancies, as well as veiled dielectric-responsive character, are instrumental in electromagnetic dissipation. Conventional methods, however, constrain their delicate constructions. Herein, an innovative alternative is proposed: carrageenan-assistant cations-regulated (CACR) strategy, which induces a series of sulfides nanoparticles rooted in situ on the surface of carbon matrix. This unique configuration originates from strategic vacancy formation energy of sulfides and strong sulfides-carbon support interaction, benefiting the delicate construction of defects-rich heterostructures in M_x_S_y_/carbon composites (M-CAs). Impressively, these generated sulfur vacancies are firstly found to strengthen electron accumulation/consumption ability at heterointerfaces and, simultaneously, induct local asymmetry of electronic structure to evoke large dipole moment, ultimately leading to polarization coupling, i.e., defect-type interfacial polarization. Such “Janus effect” (Janus effect means versatility, as in the Greek two-headed Janus) of interfacial sulfur vacancies is intuitively confirmed by both theoretical and experimental investigations for the first time. Consequently, the sulfur vacancies-rich heterostructured Co/Ni-CAs displays broad absorption bandwidth of 6.76 GHz at only 1.8 mm, compared to sulfur vacancies-free CAs without any dielectric response. Harnessing defects-rich heterostructures, this one-pot CACR strategy may steer the design and development of advanced nanomaterials, boosting functionality across diverse application domains beyond electromagnetic response.

**Supplementary Information:**

The online version contains supplementary material available at 10.1007/s40820-024-01515-0.

## Highlights

- A series of sulfides/carbon composites with sulfur vacancies-rich sulfides heterointerfaces are well-designed and developed via a simple one-pot carrageenan-assistant cations-regulated strategy.
- “Janus effect” of interfacial sulfur vacancies, which triggers strong defect-type interfacial polarization, are firstly intuitively confirmed by both theoretical and experimental investigations.
- Optimized Co/Ni-carbon composites (CAs) imbued with sulfur vacancies-rich heterointerfaces displays broad absorption bandwidth of 6.76 GHz at only 1.8 mm, compared to sulfur vacancies-free CAs without any dielectric response.

**Supplementary Information:**

The online version contains supplementary material available at 10.1007/s40820-024-01515-0.

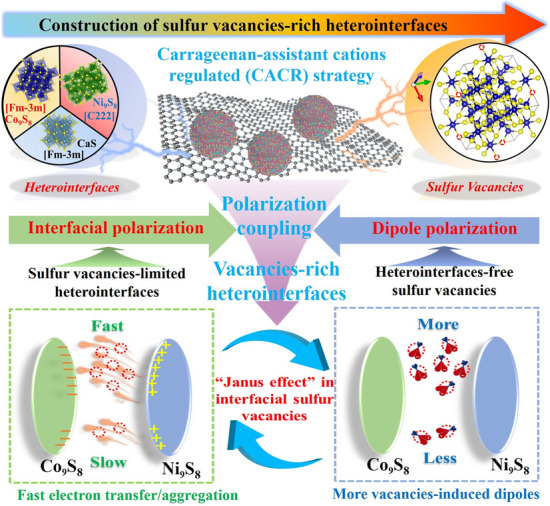

## Introduction

The extensive use of advanced emerging technologies such as wireless electronics to satisfy the growing global communication of the modern world has brought about considerable electromagnetic pollution and irreversible human health issues [1]. Electromagnetic wave absorbing materials (EMWAMs), typically involving the heterogenous dielectric absorbers, play critical roles in dissipating these unwanted radiations. Transition metal sulfides (TMSs) are emerging as promising EMWAMs because of their tailored metal components, abundant crystal structures, tunable electronic structure and easily achieved defects [2, 3]. Nonetheless, only on their own structure and properties without additives, the pure TMS always suffers from inferior intrinsic electronic conductivity and low dielectric response, resulting in limited electromagnetic wave (EMW) attenuation mechanisms and unsatisfactory absorption bandwidth [4]. Although tremendous efforts have been devoted in pursuit of satisfactory dielectric TMSs during the past few decades, yet high-efficiency TMSs featured with broader effective absorption bandwidth (EAB) are still missing with their development and remained challenging.

Typically, electron formation, transport, and aggregation at surfaces/interfaces can significantly affect dielectric loss containing polarization and conduction loss [5–7]. Building multiple-phases TMSs with ample heterostructure have been recognized as an appealing strategy for enhancing dielectric polarization loss owing to that it could provide unprecedented effects at the heterointerfaces [8, 9]. The component units of these heterostructures with different bandgaps can afford the benefits of stimulating the built-in electric field effect, which allows for fast charge transport and satisfactory unsaturated electronic configurations [10]. Benefiting from these well-designed heterostructures, opposite charges are inevitably inclined to accumulate at diverse heterointerfaces, bringing about many interfacial dipolar vibrations to dissipate electromagnetic (EM) energy [11, 12]. Various heterostructure composites, such as sulfides/sulfides [13, 14] and sulfides/carbons heterostructures [15], have exhibited excellent polarization loss because of the modulation of the effective heterointerface area and heterointerface charge density as a result of strong electronic interactions occurring on the heterointerfaces [16]. Thus, precise tailoring of heterostructures and its effect on the interface charge accumulation/consumption are highly requirable, however, still challenging. On the other side, considering that the electron concentration in TMSs is determined by the quantity of unpaired free electrons, introduction of sulfur vacancies is reported with significant improvement in intrinsic electrical conductivity [17], which endows suitable modulation and optimization of EM parameters. What’s more, these sulfur vacancies also could greatly enhance dipoles polarization by triggering abundant high-energy unsaturated active site as defect centers to provide sufficient defect-induced electric dipoles [18]. Inspired by these hypotheses, if we can design and structure the desirable multiple-phase heterointerfaces and increase their density on sulfur vacancy (i.e., defects-rich heterostructures), various dielectric attenuation or polarization coupling mechanisms (such as defects-type interfacial polarization) may be simultaneously activated so that to make a spectacular achievement on their broader absorption bandwidth and dissipation intensity. To sum up, just like the Greek two-headed Janus showing versatility in in charge of the both past and future (i.e., “Janus effect”), this unique defects-rich heterostructures also perhaps harvest the “Janus effect” in coupling both of interfacial polarization and defect-induced dipoles polarization. However, such “Janus effect” of defects-rich heterostructures is still unclear yet.

Previous attempts have revealed that chemical doping [18, 19], F^−^ regulation engineering [20], regulating crystalline phases structure [21], and constructing hierarchical structuralization [22, 23] can effectively optimize their interfaces or/and defects characteristics. Despite considerable progress, several fatal shortcomings still remain: i) The scope for improvement in electron formation/transport/aggregation at heterointerfaces of sulfides is far from sufficient that incumbers the complete release of their intrinsic dielectric activity under the applied EM field; ii) as a result of the inert surface of traditional support materials, the interfacial interactions between the supported sulfides and the matrix materials (*e.g.*, carbonaceous material) are not strong enough to well regulate the lattice structure of sulfides nanoparticles, which in turn does not guarantee their rich heterointerfaces/vacancies characteristics, especially for defects-rich heterostructures [24, 25]; iii) the lack of insights and effective strategies that could power precise and controllable heterointerfaces/defects regulations are blocking the rational develop high-efficiency TMSs with excellent EMW absorption performance [26]; iv) due to the lack of combined experimental techniques and theoretical methods, accurate identifications and clarification of the in-depth relationships among intricate atomic-level microstructures (such as vacancies and heterointerfaces) [27], charge formation/transport/aggregation characteristics and macroscopical dielectric response behaviors are still beyond reachable. More importantly, how defects-rich heterostructures rather than individual heterointerface or sulfur vacancy can be synergistically engineered to tailor their electromagnetic wave parameters and EMW absorption, based on both experimental design and theoretical calculations rather than semiempirical rules, has been rarely explored so far. Hence, the effect of the atomic-level defects-rich heterostructures on the electronic structure characteristics and EM parameters involving EMW absorption is ambiguous. Exploring a facile and reliable strategy toward manipulating defects-rich heterostructures to well manage electronic conductivity, EM parameters, as well as gaining in-depth understanding on the underlying “Janus effect” toward EM energy-consuming mechanisms are desired and of prime significance.

Herein, to resolve the aforementioned challenges, heterointerfaces and sulfur vacancies were rationally integrated to develop *ι*-carrageenan-derived porous carbon embedded with multiple sulfides heterointerfaces bearing abundant sulfur vacancies via a facile solgel transformation and pyrolysis process. This unique design concept endows the M-CAs with multiple advantages: i) Heterointerfaces originated from the competition growth of sulfides NP can cause plenty of defects (such as sulfur vacancies) and tempt the defect-induced polarization loss; ii) in return, these generated vacancies sites allow for strengthening electron accumulation/consumption ability at the heterointerface, thereby elevating the interfacial polarization by the build-in electric field effect. Importantly, density functional theory (DFT) calculations resonantly prove the significant effects of heterointerfaces, sulfur vacancies, vacancies-free heterostructures and vacancies-rich heterostructures on density of states (DOS), charge distribution structure, and the corresponding electrostatic potential as well as dipole moment for enhancing dielectric polarization loss. Consequently, the “Janus effect” of vacancies-rich sulfides heterointerfaces make Co/Ni-CAs feature with a broad absorption bandwidth of 6.76 GHz at only 1.8 mm, compared to the CAs with inferior dielectric response. In summary, constructing defects-rich heterostructures and systematically revealing the veiled mechanism can not only help understand the effect of interfacial sulfur vacancies on dielectric response but also provide a new paradigm for the design of novel broadband EMWAMs.

## Experimental Section

### Preparation of Carrageenan-M Hydrogel

The 2 wt% *ι*-carrageenan solution was prepared at 85 °C for 55 min using the magnetic stirring. Then, the 0.1 M metal salts (such as Co(NO_3_)_2_) ethanol solution was dripped into the above solution stirring under a high speed to form carrageenan-Co hydrogel at room temperature, and then washed and freezed. For comparison, carrageenan-C, carrageenan-Ni, and carrageenan-Co–Ni were prepared by using the similar procedure as the above carrageenan-Co, in which Co(NO_3_)_2_ was replaced with no metal salts, Ni(NO_3_)_2_, as well as Co(NO_3_)_2_ and Ni(NO_3_)_2_, respectively.

### Preparation of Multiple Metal Sulfides/Carbon Composites

Firstly, the freezed carrageenan-M hydrogel was transformed into carrageenan-M aerogels hydrogels by dehydrated dehydration process through freeze drying. After that, the carrageenan-M aerogels were pyrolyzed at 600 °C for 2 h in Ar atmosphere at the heating rate of 2 °C min^−1^ to obtain the M_x_S_y_/CAs (M-CAs for short). According to the difference in metal salts, the resulting samples were marked as CAs, Co-CAs, Ni-CAs, and Co/Ni-CAs.

### Characterization

The crystal structure information of samples was analyzed by X-ray diffraction (XRD, Bruker D8 DISCOVER A25, Germany), using Cu Ka X-ray radiation at a scanning rate of 2° min^−1^ in the 2θ region of 10°-80°. A CHI 660D electrochemical workstation (with a frequency of 0.1–10,000 Hzand an amplitude of 5 mV) was performed on electrochemical impedance spectroscopy (EIS). In detection, a Pt foil, a saturated calomel electrode, and Na_2_SO_4_ aqueous solution (1 M) was utilized as counter electrode, reference electrode, and electrolyte, respectively. Furthermore, the compositional, microstructure and morphology were gained from scanning electron microscope (SEM, FEI Verios G4) and a transmission electron microscope (TEM, F200X, FEI Talos, USA) equipped with an energy-dispersive X-ray (EDX) to acquire cross-sectional images and element distributions. Chemical states characterization and defects level were investigated by X-ray photoelectron spectroscopy (XPS, Kratos AXIS Ultra DLD, UK) and electron spin-resonance resonance (ESR, Bruker E500, Germany) spectra, the microwave power was 15.00 mW, and the frequency was 9.84 GHz. Finally, a vector network analyzer (Anritsu MS46322B, Japan) using a typical coaxial-line method was conducted to obtain EM parameters (complex permittivity (*ε*_r_ = *ε'*– *jε"*) and permeability (*μ*_r_ = *μ'*–*jμ"*). Before EM parameters test, all samples (33 wt%) were pressed into rings-like (*Φ*_in_ = 3.04 mm, *Φ*_out_ = 7.00 mm) in paraffin.

### Computational Methods

All of the first-principle calculations were conducted with the Kohn–Sham density functional theory (DFT) by using the Vienna *ab* initio simulation package (VASP). The convergence criteria of the energy in electronic SCF iterations and the force in ionic step iterations are 1.0 × 10^–6^ and − 0.02 eV, respectively, for geometrical optimizations, including cube, slab, and vacancy models. The generalized gradient approximation is parameterized by Perdew, Burke, and Ernzerhof (PBE) for exchange–correlation functional. The plane-wave basis set with a default kinetic energy cutoff is used to expand the valence electron wave function. The k-spacing value to generate Gamma-centered *k*-points sample mesh is 0.04 for optimization. We also calculated the sulfur vacancy formation energy by:

$$E_{{{\text{form}}}} = E_{{{\text{S - van}}}} - E_{{{\text{slab}}}} - E_{{\text{S}}}$$

where *E*_s-van_, *E*_slab_, and *E*_s_ represent the energy of S-vacancy models, corresponding slab structures, and isolated S atom. The DOS and Bader charge were calculated for the slab models including the sulfur vacancy geometries.

The DOS and charge density difference were calculated using the Cambridge Serial Total Energy Package (CASTEP) in Material Studio. The Perdew–Burke–Ernzerhof (PBE) in the generalized gradient approximation (GGA) form was used as the exchange–correlation function. The dipole moment of a single-structure cell was implemented using the Dmol3 module with the GGA and PBE exchange–correlation functional.

### Simulation of the Radar Cross Section (RCS)

RCS simulation is used to study the effects of different samples on monostatic RCS to further verify the microwave absorption performance. The far-field electromagnetic absorption simulation of a 180 × 180 cm^2^ coated metal plate (ideal conductive layer (PEC)) is obtained by RCS (− 55° ~ 55°, *f* = 10.0 GHz). The sample/PEC dual-layer square specimen was positioned on the X-O-Y plane, with linearly polarized EMWs incident from the positive *z*-axis to the negative *z*-axis and polarization propagating along the *x*-axis.

## Results and Discussion

### Construction of Sulfur Vacancies-Rich Heterostructures in M-CAs

**Fig. 1:**
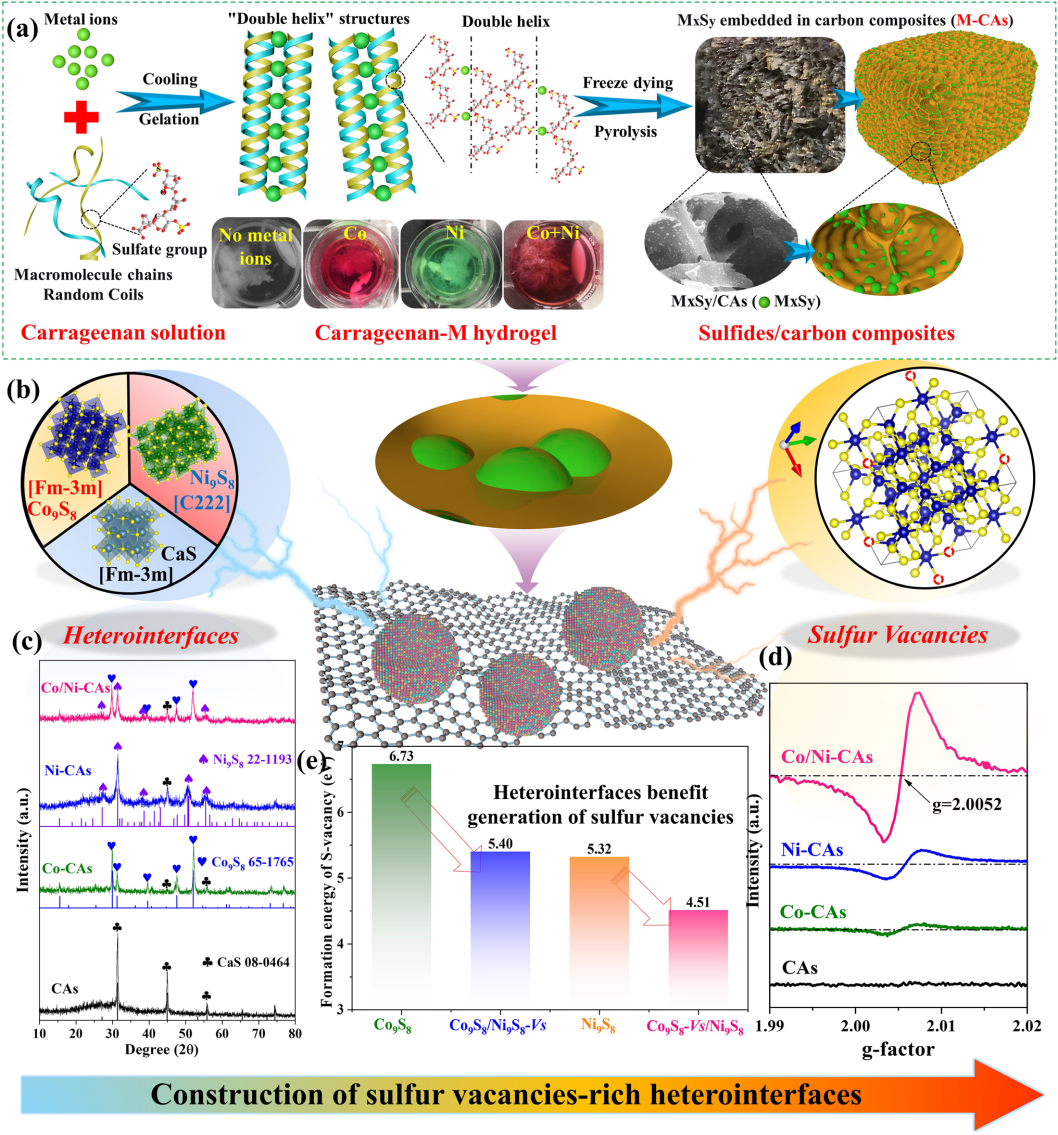
Schematic diagram of **a** preparation process and **b** construction of sulfur vacancies-rich heterointerfaces in M-CAs; **c** phase composition, **d** room temperature ESR spectra and **e** the calculated sulfur vacancies formation energy based on density functional theory (DFT) for M-CAs

A schematic illustration of the facile bottom-up synthesis approach including a solgel transformation and pyrolysis process is shown in Fig. 1a. Typically, here in the aqueous solution at a high temperature of 80 °C, the *ι*-carrageenan macromolecules chains can distribute randomly. After metal ions (M^n+^) are dipped into above solution and cooled to room temperature, the *ι*-carrageenan macromolecules will gelate with M^n+^ ions (such as Co^2+^ and Ni^2+^) leading to the formation of carrageenan-M hydrogels [28] (Fig. S1a). In this step, the M^n+^ cations are capable of forming intra-molecular bridges between the sulfate groups of iota (*ι*)-carrageenan, and meanwhile, the random coiled carrageenan macromolecules can convert into “double-helix” structures and the aggregation of them [29]. Finally, the obtained carrageenan-M hydrogels are subjected to freeze drying and subsequent pyrolysis. During the pyrolysis of carrageenan-M composites, the binding M^n+^ ions and sulfate groups transported into M_x_S_y_ nanoparticles (NPs) and carrageenan converted to porous carbon, constructing the M_x_S_y_/carbon composites (M-CAs for short) [30] (Fig. S1b–d). Further details of the experiments are provided in Experimental Section.

A point emphasized is that in the earlier literatures, the most widely used method for synthesizing metal sulfides/carbon composites always involve temperature-programmed sulfides formation, subsequent loading of sulfides on carbon source and the followed carbonization process, which are complex and energy intensive, consequently limiting the scalable manufacturing and extensive applications [31, 32]. Here we use water-soluble *ι*-carrageenan as both carbon matrix and a clean sulfur source instead of keeping them separate, making it possible to combine the stage of sulfides formation, loading and carbonization in one-pot pyrolysis process. This endows us with two advantages during pyrolysis process:

1. Diverse sulfides embedded on carbon matrix as detected by XRD in Fig. 1c that can offer abundant heterointerfaces (Fig. 1b) would be formed as a result of cations competitive reaction ability by tuning the tailored cations [9]; Herein, three different M^n+^ cations (*i.e.*, Co^2+^or Ni^2+^ or both Co^2+^ and Ni^2+^) are controlled to prepare M-CAs with sulfides heterointerfaces, while the CAs as comparison sample are also prepared by the absence of metal ions. For CAs, it is composed of CaS (PDF#08–0464) and carbon, and the former comes from the Ca^2+^ in original *ι*-carrageenan. After introducing Co^2+^, Ni^2+^, or both Co^2+^ and Ni^2+^, the diffraction peak intensity of CaS becomes inferior and a set of new peaks can be detected, corresponding to Co_9_S_8_ (PDF#65–1765) and Ni_9_S_8_ (PDF#22–1193) for Co-CAs, Ni-CAs, and Co/Ni-CAs, respectively (Fig. 1c and Table S1), which favors to generate the enriched multiple-phases heterointerfaces.
2. Due to the different vacancy formation energies of sulfides (Fig. 1e) resulting from electronegativity of the cations [33, 34], the controlled sulfur vacancies in sulfides as revealed by electron spin resonance (ESR) (Fig. 1d) could be constructed, contributing to the formation of dipoles for EMW dissipation. Notably, Co/Ni-CAs, followed by the Ni-CAs and Co-CAs, has a stronger ESR signal far more than that of CAs, which implies that multiple-phase sulfides heterostructures could benefit the creation of sulfur vacancies. To validate this conjecture, we have built Co_9_S_8_ and Ni_9_S_8_ with sulfur vacancy model (i.e., Co_9_S_8_*-Vs* and Ni_9_S_8_*-Vs*), respectively. Meanwhile, Co_9_S_8_/Ni_9_S_8_ heterointerfaces with sulfur vacancy model are also taken into consideration, in which sulfur vacancy obtained from Co_9_S_8_ is labeled as Co_9_S_8_*-Vs*/Ni_9_S_8_ and other one got from Ni_9_S_8_ is named as Co_9_S_8_/Ni_9_S_8_*-Vs*. The formation energies of sulfur vacancy structure are calculated as 6.73, 5.40, 5.32, and 4.51 eV for Co_9_S_8_, Ni_9_S_8_, Co_9_S_8_/Ni_9_S_8_*-Vs*, and Co_9_S_8_*-Vs*/Ni_9_S_8_, respectively. Evidently, the values of both Co_9_S_8_/Ni_9_S_8_*-Vs* and Co_9_S_8_*-Vs*/Ni_9_S_8_ heterointerfaces are smaller than individual Co_9_S_8_ and Ni_9_S_8_, indicating that heterointerfaces is advantageous to the enrichment of defects, well corresponding to the ESR result in Fig. 1d.

Overall, a series of M-CAs with sulfur vacancies-rich sulfides heterointerfaces are well designed and developed via a simple one-pot CACR strategy. This nanotechnology-based method involves using metal ions with different competitive reaction abilities and vacancy formation energies to finely tune the crystalline phases structure of sulfides, thereby achieving controllable generation of sulfides heterostructures and simultaneous formation of rich sulfur vacancies, which is anticipated to contribute to optimize electronic structure and the involved dielectric polarization loss.

### Morphology, Heterointerfaces and Lattice Defect Analysis of M-CAs

**Fig. 2:**
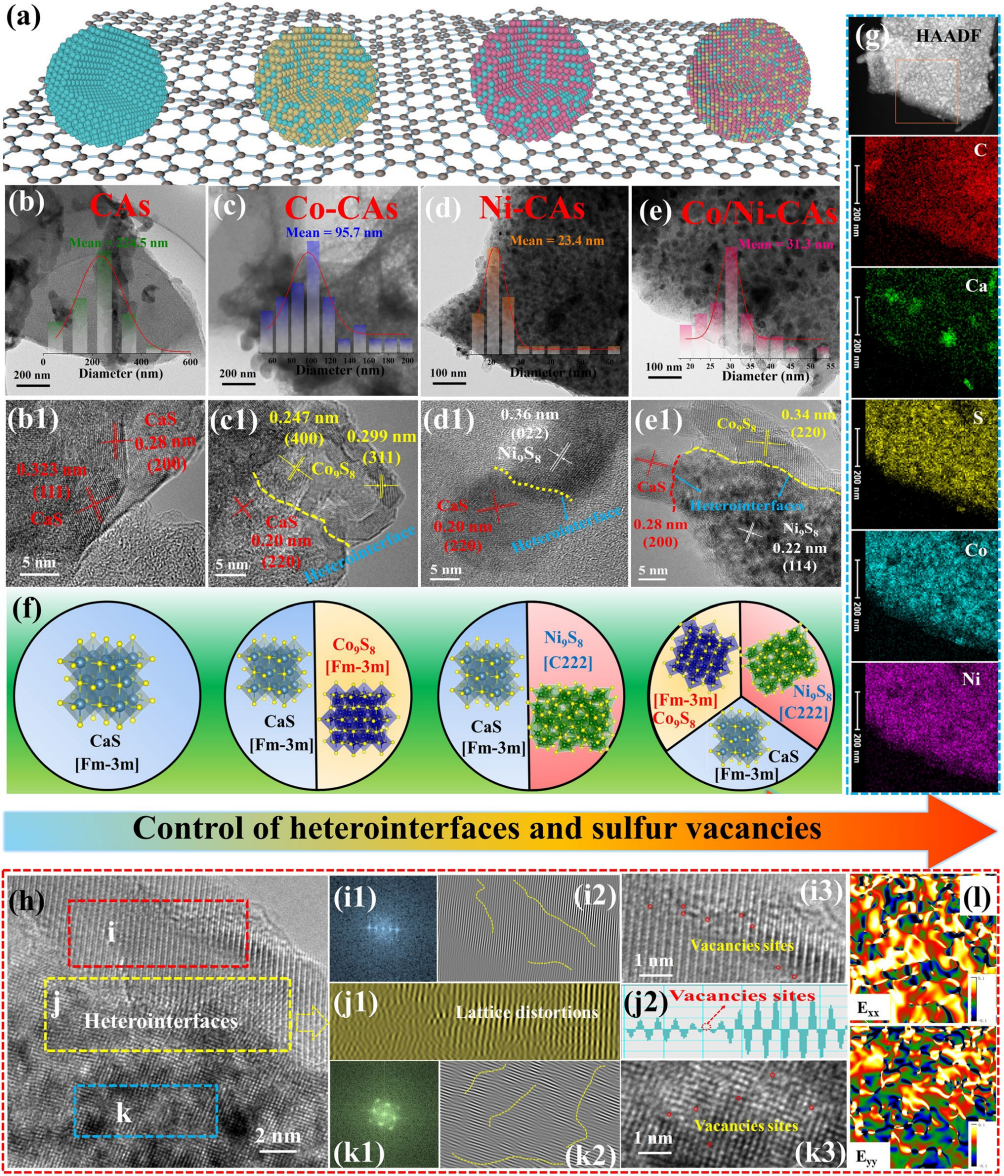
Heterointerfaces and lattice defect as well as geometric phase analysis characterization of M-CAs. **a–e** TEM images and particle size distribution; **b1–e1** HR-TEM showing the heterointerfaces in M-CAs and CAs, and **f** the corresponding schematic diagram showing heterointerfaces and sulfur vacancies could be well control by subtly regulating the metal ions; **g** TEM mapping images of Co/Ni-CAs; **h** Lattice information taken from fig. e1, displaying lattice distortion, vacancy sites and strain distributions in **i–i3** Co_9_S_8_ lattice, **k–k3** Ni_9_S_8_ lattice and **j–j2** and **l** Co_9_S_8_/Ni_9_S_8_ heterointerfaces for Co/Ni-CAs

Morphology, heterointerfaces, and lattice defects changes, as a function of metal ions, are analyzed by SEM and TEM. As delineated in (Fig. S2b–d), all M-CAs preserve the original porous configuration with pore structures and interconnecting channels, in contrast to the CAs (Fig. S2a). Figure 2a–e shows the TEM images of CAs, Co-CAs, Ni-CAs, and Co/Ni-CAs M-CAs, as well as their particle size distributions, respectively (inset in Fig. 2b, e). One can clearly see that the obtained M_x_S_y_ NPs are in situ embedded on the carbon surface without visible agglomeration. These monodisperse particle distribution may benefit interfacial polarization. Among them, abundant singly dispersed M_x_S_y_ NPs with smaller particle size are observed on M-CAs (Fig. 2c–e) especially for Ni-CA and Co/Ni-CA relative to pure CAs (Fig. 2b), which is robustly confirmed with SEM. These extensively dispersed metal particles are believed to induce local sulfides-carbon interfacial interaction and provide more active sites, which are potentially advantageous to facilitate heterointerfaces and sulfur vacancy formation [35, 36]. The detailed lattice structure information is further revealed by high-resolution transmission electron microscopy (HR-TEM). As shown in Fig. 2b1, the interplanar spacing determined to be 0.28 and 0.32 nm can be separately indexed to the (200) and (111) planes of CaS for CAs, which is consistent with that of the XRD analysis. With respect to Co-CAs, contrastingly, an interfacial zone between Co_9_S_8_ (0.24 nm, (400) plane) and CaS (0.20 nm, (220) plane) is presented in Fig. 2c1, indicating the heterointerface formation due to the introduced Co^2+^. Likewise, an obvious heterointerface has successfully constructed for Ni-CAs in Fig. 2d1. Noteworthy, benefiting from the simultaneous introduction of Co^2+^ and Ni^2+^, more obvious heterointerfaces induced by the co-existence of multi-phases, associated with the selected area electron diffraction (SAED) pattern observations (Fig. S2), are created for Co/Ni-CAs (Fig. 2e1). C, Ca, S, Co, and Ni elements are distributed throughout the Co/Ni-CAs nanosheets according to the EDS mapping results in Fig. 2g, showing the successful synthesis of ternary sulfides heterointerfaces nanospheres embedded into the porous carbons. Thereinto, the distribution of Co and Ni are homogeneous while that of Ca shows obvious aggregation. It may be attributed that due to the Ca cation coming from the original *ι*-carrageenan itself, its sulfides (CaS) may tend to accumulate into the carrageenan-derived carbon matrix itself, compared to the introduced Co and Ni cations. Considering these heterointerfaces are helpful to promote interfacial polarization, the Co/Ni-CAs may harvest stronger interfacial polarization among these M-CAs samples.

Generally, metal ions with different ionic radii, electronegativities and chemical activities inevitably bring about some structural defects and lattice mismatches during polycrystal phases structure formation in sulfuration pyrolysis process [37]. Taking the Co/Ni-CAs as example, we can see that marked mismatches (yellow dotted line) exist in the lattice stripes of local zone of heterointerfaces from Fig. 2e1, indicating a large number of lattice distortions occur in Co_9_S_8_ phase (Fig. 2i1, i2) and Ni_9_S_8_ phase (Fig. 2k1, k2) as well as Co_9_S_8_/Ni_9_S_8_ heterointerfaces (Fig. 2j1, j2). Especially, large amounts of discontinuities vacancies sites as point defects (red dotted circle) are also significantly observed in (Figs. 2j2 and S3) and around the Co_9_S_8_/Ni_9_S_8_ heterointerfaces lattices (Fig. 2i3, k3), once again confirms the highly vacancies characteristic of Co/Ni-CAs as found in the outstanding vacancy peaks in ESR (Fig. 1d). The geometric phase analysis (GPA) is further performed to reveal the defect-induced strain distribution state near the heterointerfaces in detail based on the HR-TEM images [38]. In Fig. 2l, the strain distributions of E_xx_ and E_yy_ are obtained by GPA from Fig. 2h. The emergence of dramatic strain variations with more intense tensile and compressive strains near heterointerfaces firmly supports the fact that heterointerfaces is advantageous to the enrichment of defects (including lattice distortions and sulfur vacancies). Such defects contribute to alter charge transport and trigger charge aggregation, which lead to generating spatial dipole moments to boost polarization relaxation for EMW attenuation. These results consistently demonstrate heterointerfaces and sulfur vacancies, and vacancies-rich heterointerfaces could be well controlled by subtly regulating the metal ions in this work based on CACR strategy as illustrated in Fig. 2a, f. Thereinto, in comparing to pure CAs, Co/Ni-CAs has apparent heterointerface features accompanied with highly vacancies nature. Since these abundant heterointerfaces and enriched defects can disrupt the periodicity of the lattice and modify the charge carrier transport model, they are likely gainful to reinforce electron accumulation/consumption ability and intensify dielectric polarization loss, which will be discussed in the following content in detail.

### Compositional and Interfacial Electron Interactions Information of M-CAs

XPS was employed to examine the chemical bond and chemical state of M-CAs. The survey spectra in Fig. S4a display peaks of the C, S, Ca, and metal ions (Co or/and Ni), consistent with the EDS observations and XRD analysis. The high-resolution C 1*s* spectrum is given in Fig. S4b. Aside from C = C (284.6 eV), a characteristic peak of C–S/C–O (285.6 eV) bond manifests that the M_x_S_y_ nanoparticles and carbon matrix are strongly combined, showing the active carbon support materials in this work, rather than inert surface of traditional support material. Such interfacial interactions between the supported sulfides nanoparticles and the carbon matrix materials are probably anticipated to expedite the construction of defects-rich sulfides heterostructures.

**Fig. 3:**
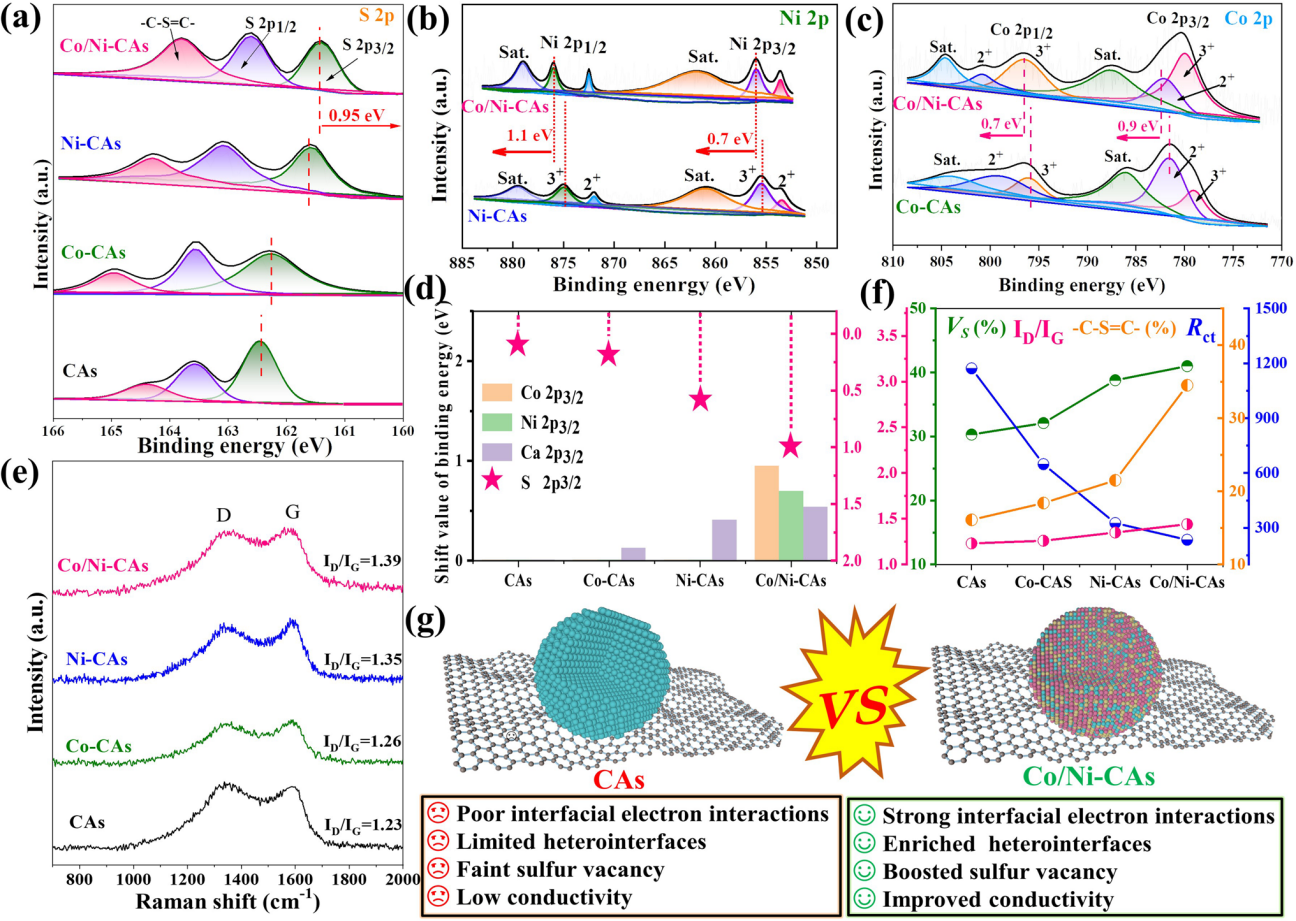
High-resolution XPS spectra of **a** S 2*p*, **b** Ni 2*p*, and **c** Co 2*p*; **d** Histogram showing the shift in binding energy values for M-CAs in comparison with CAs; **e** Raman spectra; **f** comparisons in sulfur vacancy (*Vs*),* I*_*D*_*/I*_*G*_ in Raman, –C–S = C bond in XPS spectra and charge transfer resistance (*R*_ct_), showing the structural advantage for M-CAs, especially for **g** Co/Ni-CAs relative to CAs in boosting heterointerfaces, sulfur vacancies, the resulting interfacial electron interactions and conductivity

As a main elemental, the high-resolution XPS spectra of S are shown in Fig. 3a. Three peaks at 161.4, 162.5, and 163.3 eV are assigned to S 2*p*_3/2_, S 2*p*_1/2_, and –C–S = C bond. Obviously, the binding energies of S 2*p*_3/2_ in Co-CAs and Ni-CAs exhibit negative shifts compared with CAs, while the co-introduction of both Ni and Co brings about a larger negative shift of S 2*p*_3/2_ for Co/Ni-CAs, demonstrating stronger electron acquisition of S due to the heterointerfaces construction between diverse sulfides. Meanwhile, the pronounced positive shifts indicating strong electron loss in metal atoms could also be apparently discernible for Co/Ni-CAs than other samples (Figs. 3b, c and S1c), as calculated and depicted in Fig. 3d and Table S2. These results manifest the stronger interface electron accumulation/consumption between S atom and metal atoms and indirectly prove that ample heterointerfaces construction of different sulfides NPs for Co/Ni-CAs, followed by Ni-CAs and Co-CAs than heterostructure-free CAs, which is good in line with the analysis of HR-TEM (Fig. 2b, e) and XRD (Fig. 1c). In addition, a XPS peak centered at 163.3 eV, belonging to –C–S = C bond, is related to carbon plane defects, which may arise from the strong interaction of carbon substrate and its loaded sulfides nanoparticles (NPs) during synthesis process [39]. As shown clearly in Table S3, the peak areas of –C–S = C bond are as follows: Co/Ni-CAs > Ni-CAs > Co-CAs > CAs, and this order is well consistent with *I*_*D*_*/I*_*G*_ peak intensity ratios in Raman spectra (Fig. 3e, f). These consequences suggest that more intense interfacial reaction between carbon substrate and its loaded sulfides NPs emerges for heterostructured Co/Ni-CAs compared to other samples, further straightly confirming successful formation of multiple-phases heterointerfaces as we expected. On the other hand, it was previously reported that these strong electron interactions between carbon substrate and its loaded sulfides NPs, as well as sulfides NPs themselves would like to easily trigger severe surface-disordered structures and promote the production of defect levels in the transition metal sulfides [24, 25]. Compared with pure CAs (30.3%), a larger value of sulfur vacancy concentration (*Vs*) for Co/Ni-CAs (40.9%), followed by Co or Ni-CAs (32.0% or 39.6%, respectively) are detected in Table S3 and Fig. 3a, which is in line with the tendency of ESR spectra (Fig. 1d), and is analogous to the mentioned above results of *I*_*D*_*/I*_*G*_ in Raman and -C-S = C bond in XPS spectra (Fig. 3f), further confirming our speculation. Besides, when electrons pass through these defective regions, they will be hopped or captured, therefore, to contribute to the controlled conductivity. To investigate the electronic transfer resistance (*R*_ct_), electronic impedance spectra (EIS) was then conducted. As seen from the Nyquist plots (Fig. S4d), the value of *R*_ct_ of Co/Ni-CAs show much lower than that of CAs, implying its enhanced electron conductivity (Fig. 3f). Besides contributing to improving electrical conductivity, it is well accepted that these sulfur vacancies can perform as unsaturated coordination active sites that could induce dipoles, contributing to the enhanced conduction loss and dielectric polarization for EMW attenuation spontaneously. In this regard, Co/Ni-CAs is anticipated to harvest more intense dielectric loss (including interfacial polarization and defect-induced polarization, as well as the “Janus effect” related polarization coupling) than other samples. Collectively, these quantitative results confirm the co-existence of enriched heterointerfaces and sulfur vacancies for heterostructured M-CAs, as well as the strongest interfacial electron interactions in vacancies-rich heterostructured Co/Ni-CAs compared to sulfur vacancies-free CAs (Fig. 3g). Benefiting from these merits, the EM performance and dielectric properties could be finely tuned and well optimized.

### Electromagnetic Parameters and EMW Absorption Performance of M-CAs

**Fig. 4:**
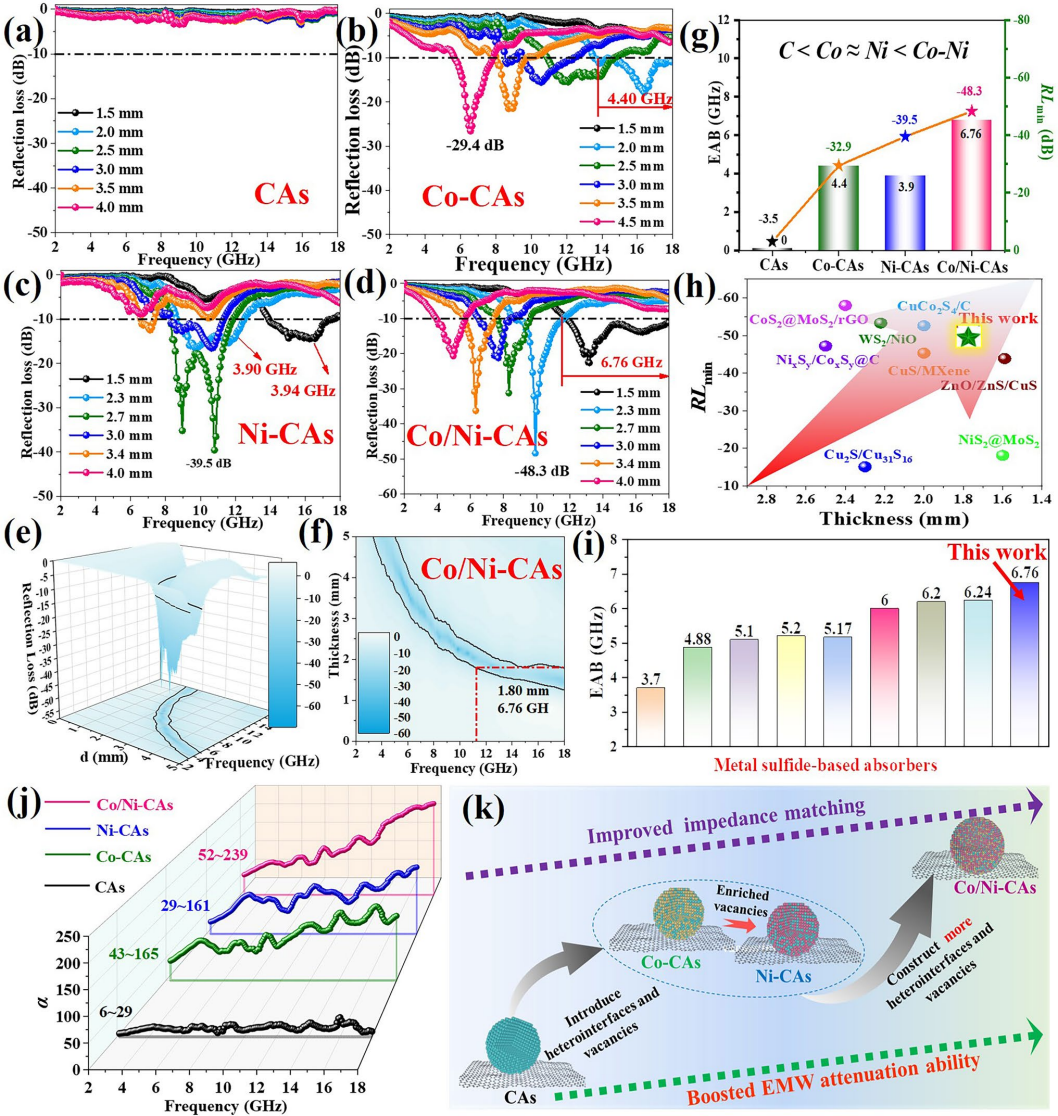
*RL* plots of** a** CAs,** b** Co-CAs, **c** Ni-CAs, and **d** Co/Ni-CAs; **e** 2D *RL* plots and **f** 3D *RL* plots for Co/Ni-CAs; **g** comparison of the EAB with the optimal thicknesses of the samples; comparison of the **h** *RL*_min_ and **i** EAB of Co/Ni-CAs to other reported metal sulfides-based absorbers; **j** attenuation constant (α) for samples; **k** schematic diagram showing the improved impedance and boosted EMW attenuation ability of M-CAs, especially for Co/Ni-CAs, relative to CAs through the well control of heterointerfaces and sulfur vacancies

The as-synthesized M-CAs with controllable defective sulfides-based NPs anchored onto the 3D carbon skeletons can result in tunable heterostructures and sulfur vacancies. This make it advantageous to modulate polarization behaviors and EMW absorption, therefore, their EMW dissipation abilities are examined at 2–18 GHz. For pure CAs, its limited heterointerfaces and faint sulfur vacancies endow it with complete EMW transmission rather than intensive absorption performance in terms of both reflection loss values (*RL*_min_) and effective absorption band (EAB), as shown in Figs. 4a and S5a. When Co^2+^ or Ni^2+^ is, respectively, introduced, profiting from the increased sulfur vacancy concentration and heterointerfaces via forming the multiple-phases sulfides, broader EABs of 4.40 and 3.90 GHz and higher *RL*_min_ of − 29.4 and − 39.5 dB, are attained for Co-CAs (Figs. 4b and S5b) and Ni-CAs (Figs. 4c and S5c) relative to CAs. This result means the sulfur vacancy and heterointerfaces are both beneficial to provoke EMW attenuation. Noted that although Ni-CAs possesses slightly higher sulfur vacancy concentration than Co-CAs as characterized by ESR in Fig. 1g and Table S3, contrastingly, it exhibits slightly smaller absorption bandwidth, proofing that individual sulfur vacancy or heterointerfaces is not sufficient on their own to bolster EMW consumption. Remarkably, owing to the synergistically improved sulfur vacancy concentration and promoted multiple-phases heterointerfaces by the co-introduction of Co^2+^ and Ni^2+^, the optimal *RL*_min_ further increases to − 48.3 dB for vacancies-rich heterostructured Co/Ni-CAs (Fig. 4d), and the EAB spreads 6.76 GHz with a relatively thin thickness of only 1.80 mm (Figs. 4e, f and S5d), conferring its strongest absorption intensity and widest bandwidth compared to other absorbers (Fig. 4g). That is, only the synergistic enhancement of both sulfur vacancy concentration and multiple-phases heterointerfaces (i.e., “Janus effect”), rather than individual sulfur vacancy or heterointerfaces, that can contribute to achieve both superior absorption capacity and effective bandwidth. As is well known, strong EMW absorption intensity and broad bandwidth with a thin matching thickness are desirably pursued for practical application in military and civilian areas. To this end, a comprehensive comparison has been conducted between the developed M-CAs and previously reported sulfides/carbon composites for EMW absorption [40–47]. It is distinct that both the strong *RL* values (Fig. 4h) and broad EAB (Fig. 4i and Table S4) could be comparable to those of other advanced absorbers. Remarkably, we can harvest such outstanding EMW absorption performance only with a low filler ratio (33 wt%) and thin matching thickness (1.80 mm), signifying its great potential for practical applications as a thin, broad and high-efficiency EMW absorber.

To thoroughly exploit the EMW absorption performance variance of the heterostructured M-CAs, their EM parameters are measured and exhibited in Fig. S6a, b. One can see that the *ε′* values can range from 3.2 to 3.1, 7.3 to 5.6, 9.2 to 6.3 for CAs, Co-CAs and Ni-CAs, respectively. And the highest *ε′* value oscillating from 12.4 to 4.9 is obtained for Co/Ni-CAs, suggesting its stronger EM storage capability. Likewise, for the imaginary part of permittivity (*ε″*) that reflects the dielectric loss ability, the Co/Ni-CAs also displays the highest *ε″* value oscillating between 3.8 and 10.1, followed by Ni-CAs (2.1–5.8) and Co-CAs (1.3–1.7), while the CAs roughly maintains a constant of about 0.1 as the frequency evenly increases. Both values of highest *ε′* and *ε″* suggest there exist strongest abilities of dielectric energy storage and loss in M-CAs, especially for Co/Ni-CAs, in comparison with CAs. The variation tendency of the permittivity is in line with the defects/heterostructures characteristic (Figs. 1d and 2) and confirms that the metal ions in our proposed carrageenan-assistant cations-regulated (CACR) strategy could indeed be used to adjust and optimize the EM parameters and dielectric properties. As for permeability, Fig. S6d, e displays curves for real part (*μ′*) and imaginary part (*μ′′*), where the former presents magnetic energy storage and the latter signifies magnetic energy loss capacity. Across the 2–18 GHz frequency range, *μʹ* values almost range from 0.8 to 1.3 and scarcely vary for M-CAs. This result means the introduction of metal ions shows a little effect on the *μ′* values. Besides, no apparent resonance peaks are visible in the imaginary permeability map, indicating inferior magnetic response for these samples. Additionally, the higher tan*δε* values rather than tan*δ*_*μ*_ of M-CAs, especially for Co/Ni-CAs, suggest the magnetic loss does not play a dominant role in EMW absorption process (Fig. S6c-f) as compared to CAs.

*Cole–Cole* plots are analyzed to dissect the origin of the dielectric losses based on Debye theory. Theoretically, a semicircle is assigned to the one polarization relaxation process (including interfacial/dipole polarization) in an *ε″* versus *ε′* plot while the long smooth line is linked to the conduction loss. Clearly, no visible long lines in M-CAs and CAs excludes the contribution of conduction loss for these four samples. This phenomenon can be attributed to the disordered structure of carrageenan-derived carbon matrix under the low synthetic temperature (600 °C in this work) that would reduce conductivity. Besides, the strong interaction between carbon matrix and its loaded sulfides NP further aggravates the disordered degree to reduce conductivity. This speculation can be confirmed by the very low *ε″* value and the poor EMW absorption performance in pure CAs. For CAs, we hardly find any observable semicircles in Fig. S7a, b although there exist some carbon/CaS heterointerfaces in its crystal lattices (Fig. 2b1), suggesting the limited carbon/CaS heterointerfaces are not enough induce apparent interfacial polarization/dipole polarization. on the contrary, a few distorted semicircles are emerged for Co-CAs (Fig. S7c), and the introduced heterointerfaces (Fig. 2c1) and sulfur vacancies (Fig. 1d) can account for such generations of interfacial polarization and defect-induced dipole polarization. Moreover, much distorted *Cole–Cole* trajectories in Fig. S7d suggest the improved Debye polarization relaxation processes for vacancies-rich Ni-CAs compared to Co-CAs. On the one hand, the smaller sizes distribution of sulfides NPs (95.7 nm of Co-CAs (Fig. 2c) *vs* 23.4 nm of Ni-CAs (Fig. 2d) may provide larger interfacial contact area between CaS and Ni_9_S_8_, as well as between these sulfides NPs and carbon matrix, thereby boosting interfacial polarization. On the other hand, the lower sulfur vacancies formation energy of Ni_9_S_8_ (i.e., 5.32 eV) means its higher sulfur vacancies concentration for Ni-CAs in comparison with that of Co_9_S_8_ (i.e., 6.73 eV) for Co-CAs (Fig. 1d, e), which benefits defect-induced dipole polarization. Notably, continuing to introduce simultaneously Ni and Co ions for Co/Ni-CAs, the rich polarization relaxation processes are signally revealed by the much more *Cole–Cole* semicircle with larger diameter (Fig. S7a, d) compared to Ni-CAs, Co-CAs, let alone to sulfur vacancies-free CAs. This finding indicates stronger interfacial/dipole polarization occur stimulated by both enhancements of the enriched heterointerfaces and sulfur vacancies as we found in HR-TEM (Fig. 2h–k3) and ESR (Fig. 1d). Of note, the multiple fluctuation peaks in *ε,″* which are relevant to the presence of polarization relaxation, are also obtained for vacancies-rich heterostructured Co/Ni-CAs than other samples (Fig. S6b), further proving our analysis.

It is well accepted that these polarization relaxations will reinforce the overall dissipation capabilities estimated by an attenuation constant (*α*) [48]. In Fig. 4j, the values of three M-CAs far away exceed that of CAs (from 6 to 29), suggesting the introduced metal ions implement a positive role in boosting EMW dissipation. It is worth mentioning that the Co-CAs (from 43 to 165) has a comparable value to Ni-CAs (from 29 to 161) in spite of the latter showing the higher sulfur vacancies. Once again, it confirms the individual sulfur vacancy or heterointerface is not enough on their own to strengthen attenuation capability. Encouragingly, thanks to the synergetic improvement in heterointerface and sulfur vacancies, the largest value from 52 to 239 for vacancies-rich heterostructured Co/Ni-CAs implies its superior attenuation capacity among these M-CAs. The impedance matching, as another key factor, is also taken into consideration as shown in Fig. S8. The larger area between *Z* = 0.8 (black solid lines) and *Z* = 1.2 (red solid lines) indicates the better impedance matching characteristic of M-CAs than CAs, especially for Co/Ni-CAs, being good accord with the *Cole–Cole* plots and attenuation constant (*α*). The aforementioned analysis validates the strengths of metal ions in simultaneously modulating heterostructures and defect level, which is conductive to substantial enhancement of dielectric parameters, attenuation ability and impedance matching for EMW absorption performance, as presented in Fig. 4k.

### Theoretical Calculation Analysis and EMW Loss Mechanism

To gain in-depth insight into the vital role of constructed heterostructures and sulfur vacancies in improving dielectric loss, the charge distribution structure and the corresponding DOS were plotted based on DFT calculations (Figs. S9 and 5). The associated EMW loss mechanism in the Co/Ni-CAs is elucidated based on the aforementioned discussion in Fig. 6a–c.

#### Heterointerfaces and the Related Interfacial Polarization

**Fig. 5:**
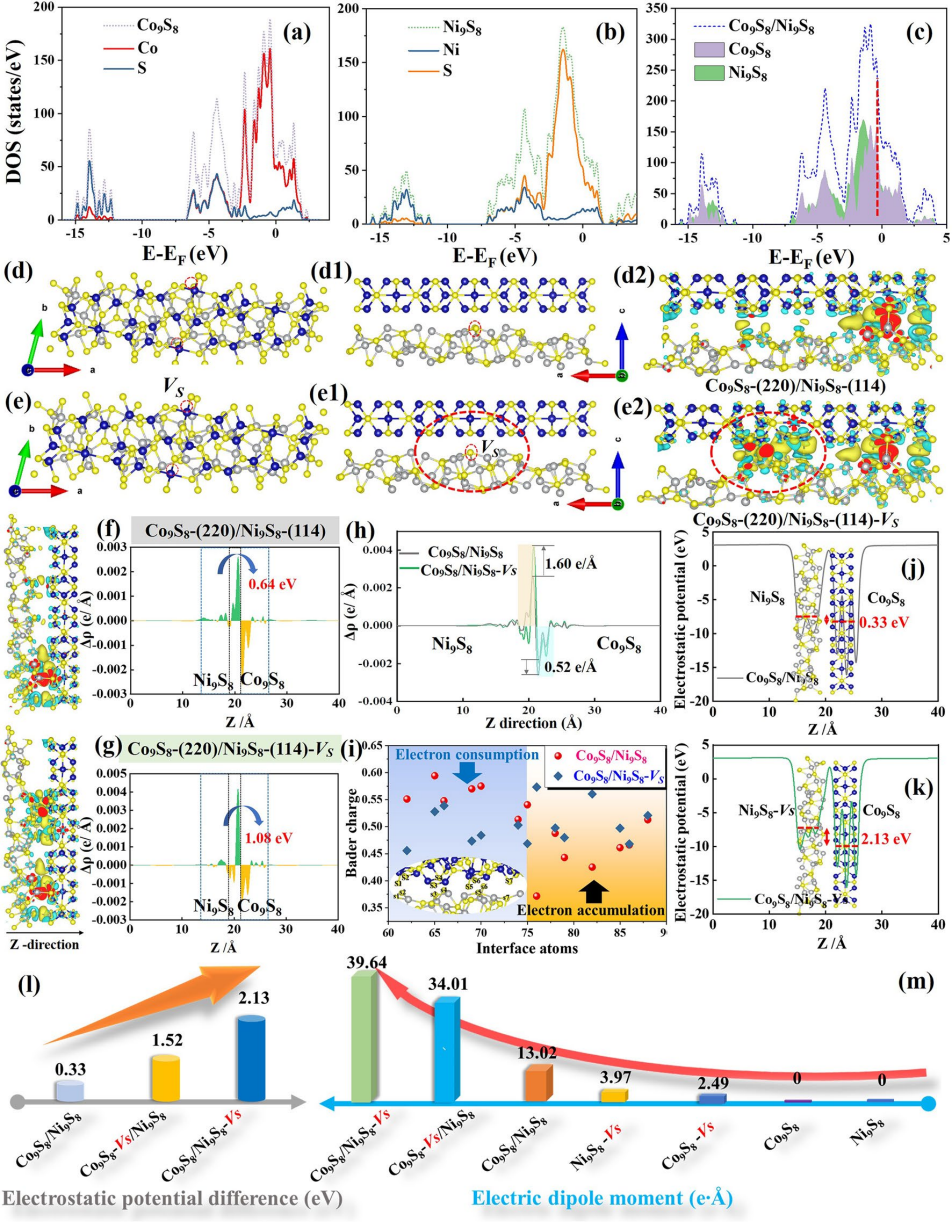
Calculated DOS of the **a** Co_9_S_8_, **b** Ni_9_S_8_, and **c** Co_9_S_8_/Ni_9_S_8_ heterointerface; front and top view as well as the charge density difference of **d–d2** Co_9_S_8_/Ni_9_S_8_ and **e-e2** Co_9_S_8_/Ni_9_S_8_-*Vs* models; the planar-averaged charge density difference along the Z direction *Δρ*(z) of **f** Co_9_S_8_/Ni_9_S_8_ and **g** Co_9_S_8_/Ni_9_S_8_-*Vs* heterointerfaces, and **h** their comparison; **i** Bader charge of interfacial atoms; electrostatic potential of **j** Co_9_S_8_/Ni_9_S_8_ and **k** Co_9_S_8_/Ni_9_S_8_-*Vs* heterointerfaces; comparisons in **l** electrostatic potential and **m** calculated dipole moment, showing the improved dielectric polarization stemmed from the sulfur vacancies and heterointerfaces

As illustrated in Fig. 5b, the electron DOS indicates the targeted Co_9_S_8_, Ni_9_S_8_ and Co_9_S_8_/Ni_9_S_8_ heterointerfaces all show the nature of the conductor, which helps the transfer of electrons due to the introduction of metal ions. Thereinto, in comparison with Co_9_S_8_ (Fig. 5a) and Ni_9_S_8_ (Fig. 5b), the electronic states of Co_9_S_8_/Ni_9_S_8_ heterointerfaces at the Fermi energy level are noticeably much higher (Fig. 5c). It implies that the abundant Co_9_S_8_/Ni_9_S_8_ heterointerfaces in the Co/Ni-CAs can greatly make the energy band denser, and further boost the charge transfer than those of Co-CAs and Ni-CAs [49]. Consequently, an increase in the accelerated interfacial charge transport improves its electrical conductivity and benefits the modulation and optimization of EM parameters, which coincides with the results shown in Figs. 3f and S6b, respectively.

**Fig. 6:**
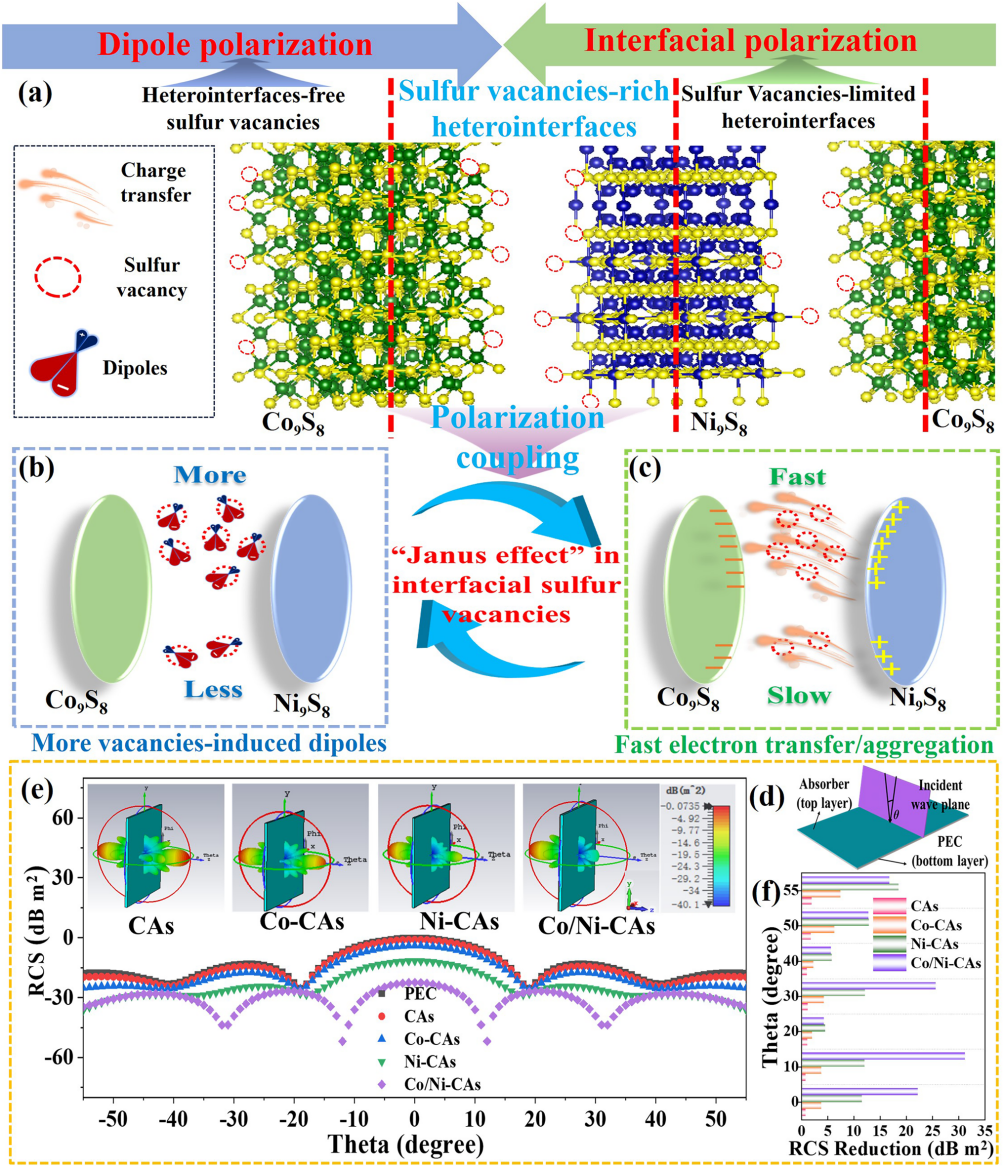
Schematic illustration of EMW absorption mechanisms of defects-type interfacial polarization (i.e., polarization coupling). **a** Co_9_S_8_/Ni_9_S_8_ with different heterointerfaces and vacancies; **b** interfacial vacancies-induced more dipoles and responding dipole polarization for Co/Ni-CAs with sulfur vacancies-rich heterointerfaces. **c** Fast electrotransfer/aggregation at heterointerfaces and responding interfacial polarization; RCS results: **d** RCS mode, **e** RCS values of PEC and PEC coated with M-CAs (insets are the responding 3D radar wave scattering signals), **f** RCS reduction values of CAs, Co-CAs, Ni-CAs, and Co/Ni-CAs

To more intuitively analyze the interface effect of the Co_9_S_8_/Ni_9_S_8_ heterointerfaces, we hence simulate the charge density difference of the heterointerface between (220) of Co_9_S_8_ and (114) of Ni_9_S_8_ as shown in Fig. 5d–d2. The calculated results reveal there is obvious imbalanced charge density distribution in the Co_9_S_8_(220)/Ni_9_S_8_(114) heterostructure (Fig. 5d2), and significant charge migration happens from Ni_9_S_8_(114) with negative charge in blue to Co_9_S_8_(220) with positive charge in yellow (where irregular yellow and blue regions correspond to the aggregation and dispersion of electrons, respectively). Theoretically, these uneven distributions of positive and negative charges will result in a large number of electric dipoles at the heterointerfaces to promote interfacial polarization under the action of alternating electric field (as shown in Fig. 6c), accompanied by EMW energy dissipation. In this regard, the interfacial polarization loss of Co/Ni-CAs is greatly enhanced due to its ample heterointerfaces compared to Ni-CAs and Co-CAs, let alone to CAs.

#### Sulfur Vacancies and the Related Defect-Induced Polarization

Furthermore, as previously mentioned, the strong electron interactions between carbon substrate and its loaded sulfides NPs, as well as the lattice deficiencies and heterogeneous interfaces derived from the competition growth of these loaded sulfides NPs themselves, will inevitably bring about the flourishing sulfur vacancies sites. To verify the contribution of sulfur vacancies to electronic structure and charge distribution of sulfides, DFT calculation is implemented. First, the tendency of sulfur vacancy formation is previously discussed (Fig. 1e), which demonstrates that the sulfur vacancies surrounding Co_9_S_8_(220)/Ni_9_S_8_(114) heterointerfaces have a lower formation energy than those surrounding individual Co_9_S_8_(220) or Ni_9_S_8_(114), confirming the crystal structure of Co_9_S_8_(220)/Ni_9_S_8_(114)-*Vs* and Co_9_S_8_(220)*-Vs*/Ni_9_S_8_(114) which are preferable in this research.

For perfect Co_9_S_8_ or Ni_9_S_8_, the charge distribution is relatively uniformly distributed (Fig. 5d1, e1). However, taking Co_9_S_8_(220)/Ni_9_S_8_(114)-*Vs* for instance, the introduction of one sulfur vacancy in Ni_9_S_8_ makes electrons delocalize at the vacancy sites and flow into nearby atomic region (the dash red circle in Fig. 5e2), which will result in the symmetry breaking of negative/positive charge centers [50]. As a result, a local electric field could be established to form a permanent dipole at the vacancy sites (as illustrated in Fig. 6b). It is well known that these dipoles will oscillate under the external EM field to enhance the defect-induced polarization loss [51]. In consideration of the difference in sulfur vacancies (Fig. 1d), these introduced sulfur vacancies of Co-CAs and Ni-CAs are salutary to enhance their defect-induced polarization losses than that of CAs [52].

#### Sulfur Vacancy-Rich Heterointerfaces Improve Polarization Coupling

Firstly, differing from sulfur vacancies-free heterointerfaces, vacancy-rich heterointerfaces can enable stronger interfacial electron accumulation/consumption by the enhanced build-in electric field (Fig. 6a). As shown in Fig. 5e2, Co_9_S_8_/Ni_9_S_8_ heterostructure with sulfur vacancy (labeled as Co_9_S_8_/Ni_9_S_8_-*Vs*) possesses more obvious difference of charge distribution at the interfaces, compared to the Co_9_S_8_/Ni_9_S_8_ heterostructure Fig. 5d2. Such stronger interfacial electron accumulation/consumption of Co_9_S_8_/Ni_9_S_8_-*Vs* than Co_9_S_8_/Ni_9_S_8_ is further revealed by the relevant planar-averaged electron density difference along the *Z* direction (Fig. 5f–h), signifying that sulfur vacancies can bring about a strong interfacial interaction [53], which is good line with XPS analysis (Fig. 3d). To obtain further understanding on this phenomenon, the Bader charge of interfacial S atoms in Co_9_S_8_/Ni_9_S_8_-*Vs* is then analyzed (Fig. 5i). It is clear that a decreasing trend of Bader charges of all interfacial S atoms occurs at the Ni_9_S_8_ (114) side (labeled as S1-S6), whereas an increasing inclination is observed for those of all the S atoms at the Co_9_S_8_ (220) side (labeled as s1-s6). Such result will demonstrate that the presence of S vacancies can enhance electron accumulation/consumption ability of interfacial S atoms. This process of self-driven charge redistribution at heterointerface will induce a built-in E-field, which is beneficial to facilitate improvement of interfacial polarization [54] (Fig. 6c). To gain a clearer insight into the strong interface effect induced by sulfur vacancy, we calculate the electrostatic potential of Co_9_S_8_/Ni_9_S_8_ and Co_9_S_8_/Ni_9_S_8_-*Vs*. As illustrated in (Fig. 5j), the difference of electrostatic potential between Co_9_S_8_ and Ni_9_S_8_ is largely heightened from 0.33 to 2.13 eV due to the introduced sulfur vacancy (Fig. 5k, l and Table S5). Consequently, the larger number of electron transfer, indicated by Bader charge with a semi-quantitative analysis [55], is harvested for Co_9_S_8_/Ni_9_S_8_-*Vs* (1.08 eV) compared to Co_9_S_8_/Ni_9_S_8_ (0.64 eV) (Fig. 5f, g), setting a fast electron transfer path and boosting interfacial polarization.

Secondly, apart from improving the migration rate of charges, these sulfur vacancies-rich heterointerfaces are able to induct local asymmetry of electronic structure to evoke much larger dipole moment, in comparison with sulfur vacancy with inferior heterointerfaces. To better understand the polarization effect arising from sulfur vacancies-rich heterointerfaces, the dipole moment is then calculated. Through Fig. 5m, it can be observed that the dipole moment of Co_9_S_8_/Ni_9_S_8_-*Vs* system is calculated as 39.64 e Å, which is three and ten time higher than those of sulfur vacancies-free heterointerfaces (i.e., Co_9_S_8_/Ni_9_S_8_, 13.02 e Å) and heterointerfaces-free sulfur vacancies (i.e., 2.49 e Å of Co_9_S_8_-*Vs* and 3.97 e Å of Ni_9_S_8_-*Vs*), respectively. Normally, the larger dipole moment can induce the stronger polarization loss and vice versa. This result means that the interfacial sulfur vacancies, rather than heterointerfaces-free sulfur vacancies or sulfur vacancies-free heterointerfaces, play more significant role in yielding dipoles, leading to an intense defect-induced polarization (Fig. 6b). Such improved performances are also found in the Co_9_S_8_-*Vs*/Ni_9_S_8_ (Fig. S10) heterointerfaces, once again confirming our above analysis.

Ultimately, polarization coupling of both interfacial and defect-induced polarization is effectively achieved via synergize heterointerfaces and sulfur vacancies in “Janus effect” of interfacial sulfur vacancies. That is, sulfur vacancy-rich heterointerfaces trigger strong defect-type interfacial polarization to enhance the EM energy consumption.

It should be emphasized that there is obvious difference among interfacial polarization, defect-induced polarization and defects-type interfacial polarization (i.e., polarization coupling), as shown in Fig. 6a–c. Normally, interfacial polarization comes from electrons aggregation at heterointerfaces, whereas defect-induced polarization is related to unbalanced distribution of local charge centers induced by the defects (such as sulfur vacancies). However, unlike these two polarizations, defects-type interfacial polarization closely depends on both heterointerfaces and sulfur vacancies, especially for the interfacial sulfur vacancies based on the strong coupling of heterointerfaces–vacancies interaction in “Janus effect” of sulfur vacancy-rich heterointerfaces (Fig. 6b, c). In other words, defects-type interfacial polarization is the effective coupling of interfacial polarization and defect-induced polarization due to the emergence of defect-rich heterostructures, which unfortunately has yet been explored based on both experimental design and theoretical calculations rather than semiempirical rules so far.

### Radar Cross-Section Simulation Results

To simulate the EMW absorption situation in the actual far-field environment, radar cross-section (RCS) distributions of the perfect conductive plate (PEC), as well as PEC covered with M-CAs, were attained using the simulation software CST and the RCS simulated model (Fig. 6d). In the incident angle-dependent RCS curves shown in Fig. 6e, Co/Ni-CAs coating displays the minimum RCS values, with RCS values below − 10 dB m^2^ as the angle changes from − 55° to 55°, and reaches − 51.89 dB m^2^ at 12°. The inset images of Fig. 6e exhibit the 3D reflected signals of samples-coated PEC, where all absorbers have similar shapes of 3D radar wave scattering signals but different intensities. In particular, the Co/Ni-CAs coating shows weaker scattering signals compared with other samples, meaning Co/Ni-CAs is well suited for EM energy dissipation. Moreover, the RCS reduction values, gained by subtracting the RCS values of the PEC from those of absorbers-coated PEC, are illustrated in Fig. 6f. As can be seen, the Co/Ni-CAs almost possess the strongest RCS reduction ability. This indicates defects-rich heterostructured Co/Ni-CAs coating has excellent inbuilt EM energy dissipation property than sulfur vacancies-free CAs even without a PEC layer, consistent with the experimental *RL* performance (Fig. 4g, h) and RCS result (Fig. 6e).

## Conclusions

In this work, carrageenan-assistant cations-regulated strategy is proposed for the first time, to induct serial monodisperse sulfides nanoparticles rooted into carbon matrix with adjustable polycrystalline phases heterointerfaces and atom vacancies, thereby achieving the delicate construction of defects-rich heterostructures. These produced vacancies are innovatively disclosed to reinforce electron accumulation/consumption ability by the build-in electric field and, more importantly, evoke simultaneously large dipole moment by breaking local symmetry of the electronic structure, ultimately leading to strong polarization coupling. Noted that such above finding of “Janus effect” in interfacial sulfur vacancies and the corresponding defects-type interfacial polarization are firstly revealed as far as we known. More significantly, the in-depth underlying mechanism for the enhanced polarization coupling in the M-CAs is clearly deciphered by the density functional theory in terms of heterointerfaces, sulfur vacancies and sulfur vacancies-rich heterostructures. As a result, the optimized Co/Ni-CAs imbued with sulfur vacancies-rich sulfides heterointerfaces display a broad absorption bandwidth of 6.76 GHz at only 1.8 mm. This comprehensive investigation of the relationships between heterointerfaces and sulfur vacancies as well as defects-rich heterostructures on synergistically elevating dielectric response opens up new prospects for developing high-efficiency EMW absorbing materials from atom-scale view, offering a generic nanotechnology-based approach to achieving controllable heterostructures design in sulfides for the targeted applications.

## Supplementary Information

Below is the link to the electronic supplementary material.

Supplementary file1 (DOCX 2365 KB)
