# Correction: Defects-Rich Heterostructures Trigger Strong Polarization Coupling in Sulfides/Carbon Composites with Robust Electromagnetic Wave Absorption

**DOI:** 10.1007/s40820-024-01608-w

**Published:** 2024-12-16

**Authors:** Jiaolong Liu, Siyu Zhang, Dan Qu, Xuejiao Zhou, Moxuan Yin, Chenxuan Wang, Xuelin Zhang, Sichen Li, Peijun Zhang, Yuqi Zhou, Kai Tao, Mengyang Li, Bing Wei, Hongjing Wu

**Affiliations:** 1 School of Physics, Xidian University, Xi’an, 710071 People’s Republic of China; 2 School of Advanced Materials and Nanotechnology, State Key Discipline Laboratory of Wide Band Gap Semiconductor Technology, Xidian University, Xi’an, 710071 People’s Republic of China; 3 School of Microelectronics, Xidian University, Xi’an, 710071 People’s Republic of China; 4 School of Telecommunication Engineering, Xidian University, Xi’an, 710071 People’s Republic of China; 5 MOE Key Laboratory of Material Physics and Chemistry Under Extraordinary, School of Physical Science and Technology, Northwestern Polytechnical University, Xi’an, 710072 People’s Republic of China; 6 The Ministry of Education Key Laboratory of Micro and Nano Systems for Aerospace, School of Mechanical Engineering, Northwestern Polytechnical University, Xi’an, 710072 People’s Republic of China

**Correction to: Nano-Micro Lett. (2025) 17:24** 10.1007/s40820-024-01515-0

Following publication of the original article [1], the authors reported the author list needed to be updated because the last three author names were duplicated.

The correct author list has been provided in this Correction. The original article [1] has been corrected.
