# Supplementary material for: Defects-Rich Heterostructures Trigger Strong Polarization Coupling in Sulfides/Carbon Composites with Robust Electromagnetic Wave Absorption
Source: Nanomicro Lett. 2024 Sep 27;17:24. doi: 10.1007/s40820-024-01515-0 (PMC11436618; doi:10.1007/s40820-024-01515-0)
Supplement: Supplementary file 1 — Supplementary file1 (DOCX 2365 KB) [file 40820_2024_1515_MOESM1_ESM.docx]

Supporting Information for

**Defects-Rich Heterostructures Trigger Strong** **Polarization Coupling in Sulfides/Carbon Composites with Robust** **Electromagnetic Wave Absorption**

Jiaolong Liu^1^, SiYu Zhang^1^, Dan Qu^1^, Xuejiao Zhou^2^, Moxuan Yin^3^, Chenxuan Wang^3^, Xuelin Zhang^4^, Sichen Li^2^, Peijun Zhang^1^, Yuqi Zhou^1^, Mengyang Li^1,^*, Bing Wei^1,^*, Hongjing Wu^5,^*

^1^ School of Physics, Xidian University, Xian 710071, P. R. China

^2^ School of Advanced Materials and Nanotechnology, State Key Discipline Laboratory of Wide Band Gap Semiconductor Technology, Xidian University, Xian 710071, P. R. China

^3^ School of Microelectronics, Xidian University, Xian 710071, P. R. China

^4^ School of Telecommunication Engineering, Xidian University, Xian 710071, P. R. China

^5^ MOE Key Laboratory of Material Physics and Chemistry under Extraordinary, School of Physical Science and Technology, Northwestern Polytechnical University, Xi’an 710072, P. R. China

*Corresponding authors. E-mail: [limengyang@xidian.edu.cn](mailto:limengyang@xidian.edu.cn) (Mengyang Li), [bwei@xidian.edu.cn](mailto:bwei@xidian.edu.cn) (Bing Wei); [wuhongjing@nwpu.edu.cn](mailto:wuhongjing@nwpu.edu.cn) (Hongjing Wu)

**Supplementary Figures and Tables**


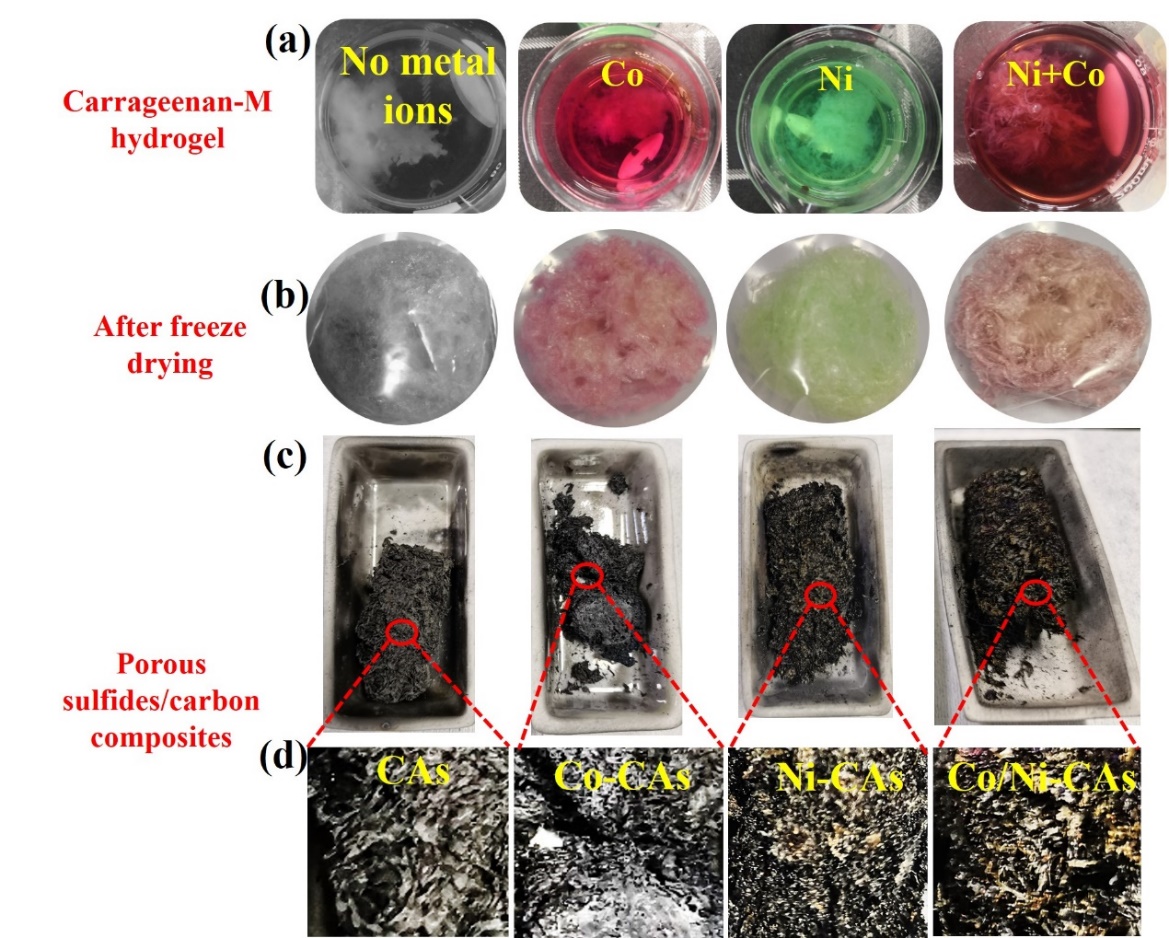


**Fig. S1** Digital photographs of (**a**) carrageenan-M hydrogel, (**b**) after freeze drying; and (**c, d**) porous sulfides/carbon composites

**
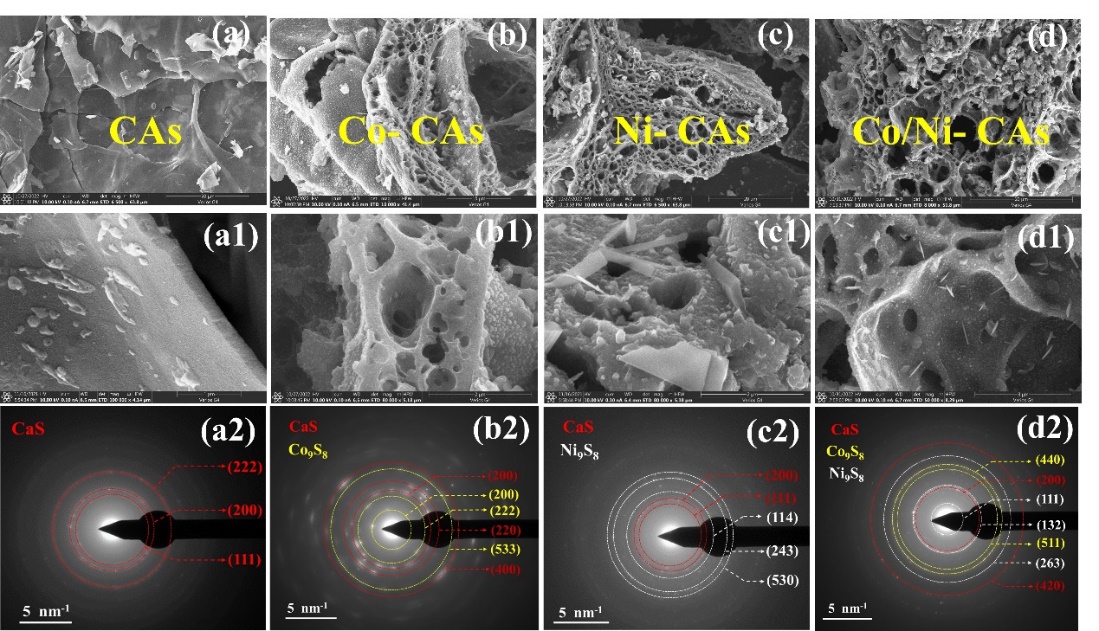
**

**Fig. S2** SEM and SAED images for (**a-a2**) CAs, (**b-b2**) Co-CAs, (**c-c2**) Ni-CAs, and (**d-d2**) Co/Ni-CAs


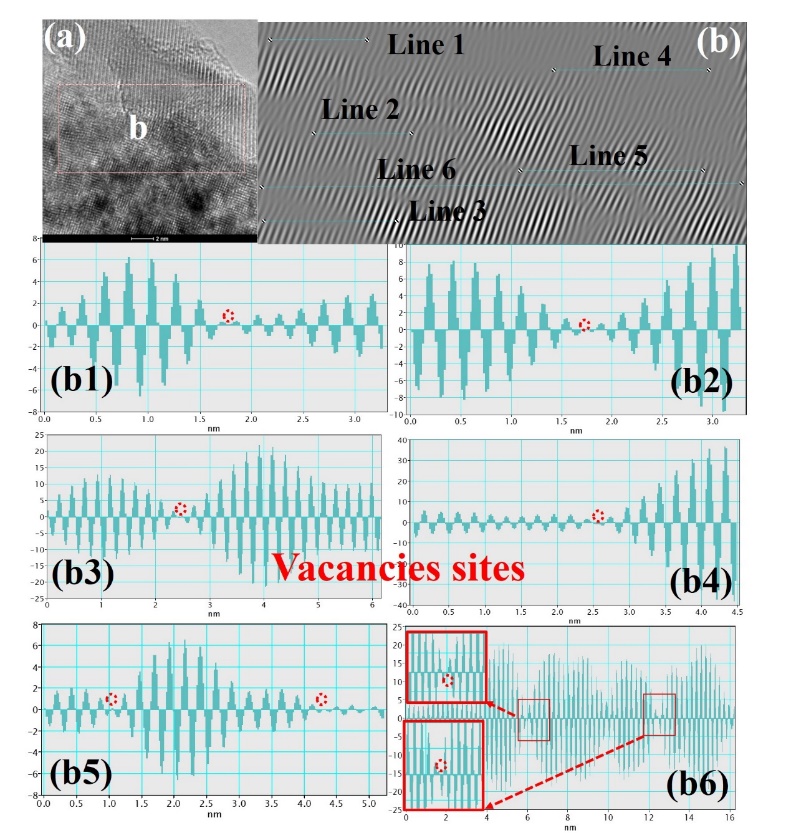


**Fig. S3** (**a**) TEM images of Co/Ni-CAs; (**b**) Lattice information taken from the figure (**a**), showing that a number of (**b1-b6**) vacancy sites exist in Co_9_S_8_/Ni_9_S_8_ heterointerfaces for Co/Ni-CAs

**
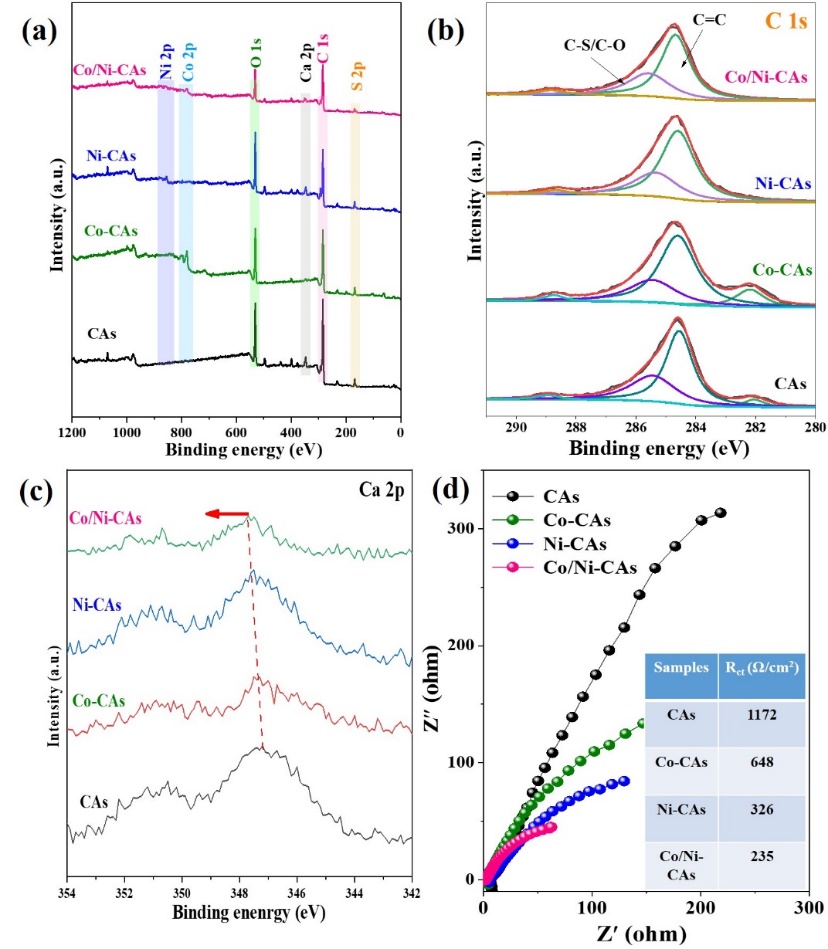
**

**Fig. S4** XPS spectra of (**a**) survey, (**b**) C 1s and (**c**) Ca 2p, and (**d**) Nyquist plots for M-CAs and CAs

**
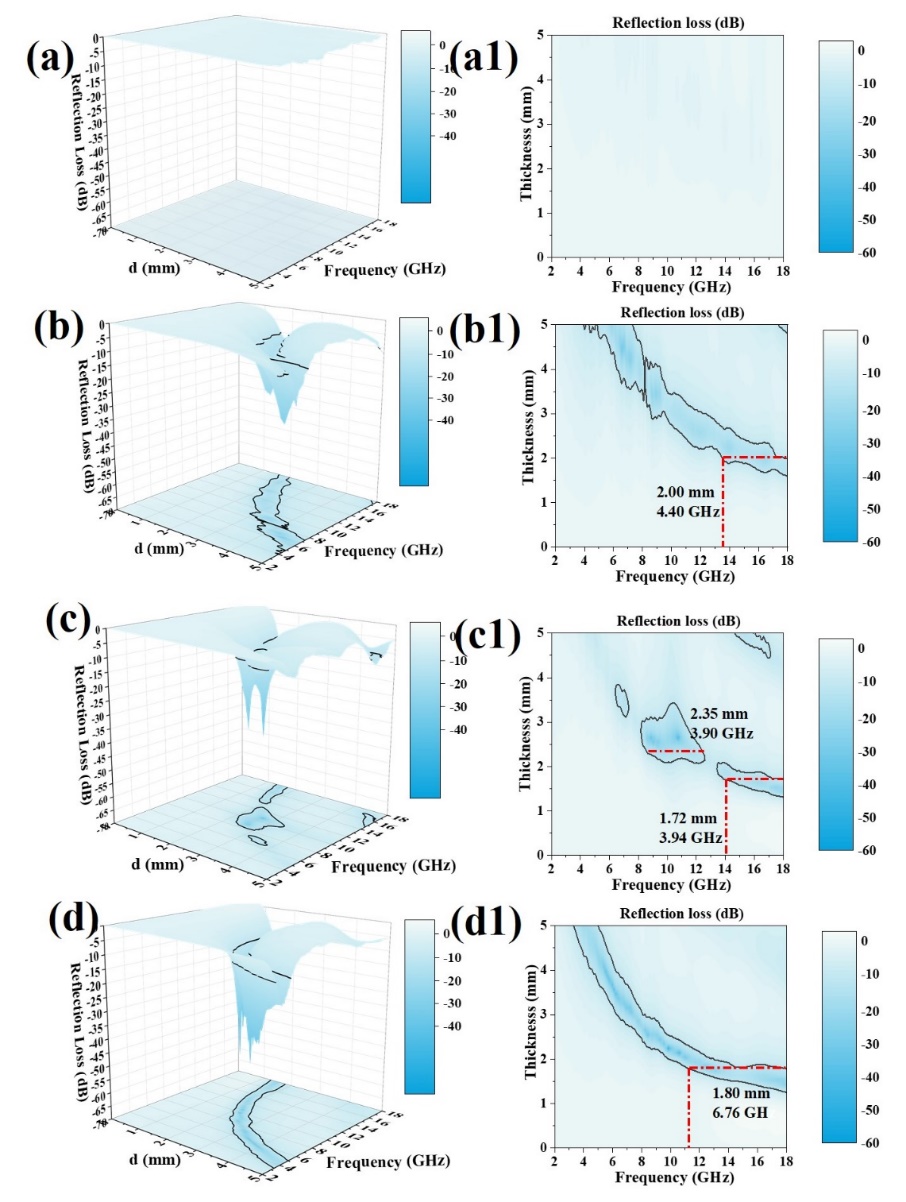
**

**Fig. S5** EMW absorption performance of samples. (**a, a1**) CAs; (**b, b1**) Co-CAs; (**c, c1**) Ni-CAs; (**d, d1**) Co/Ni-CAs


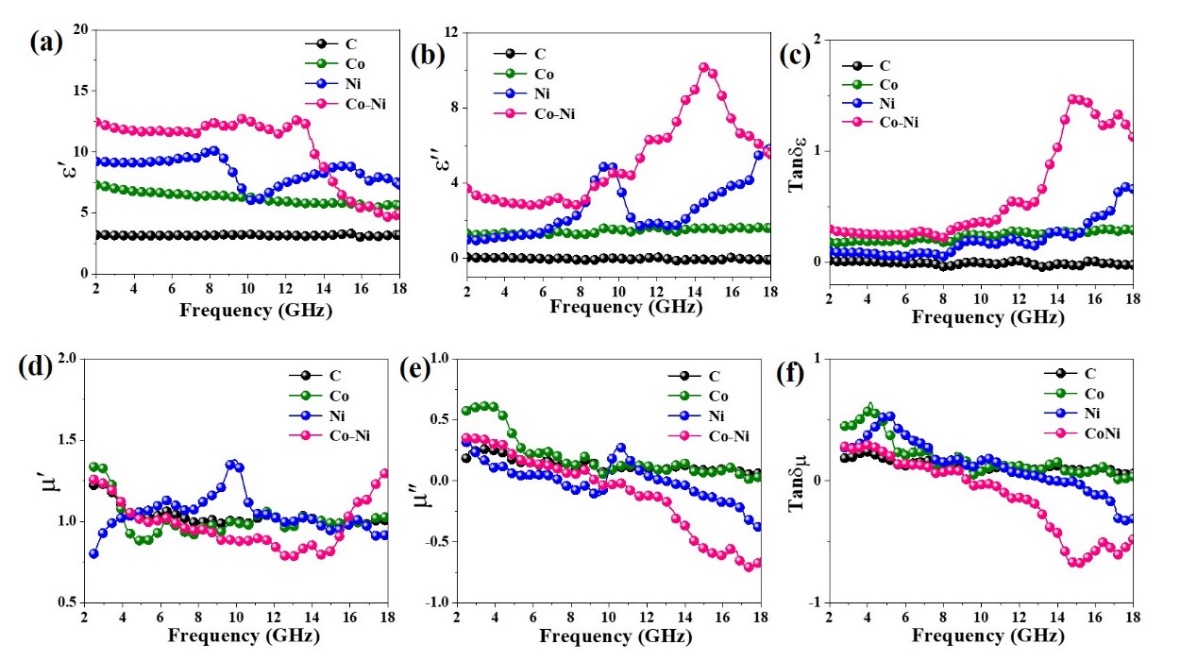


**Fig. S6** EM parameters of samples. (**a**) *ε′*, (**b**) *ε*″, (**c**) Tanδ*_ε_*, (**d**) *μ′*, (**e**) *μ*″, and (**f**) Tanδ*_μ_*

**
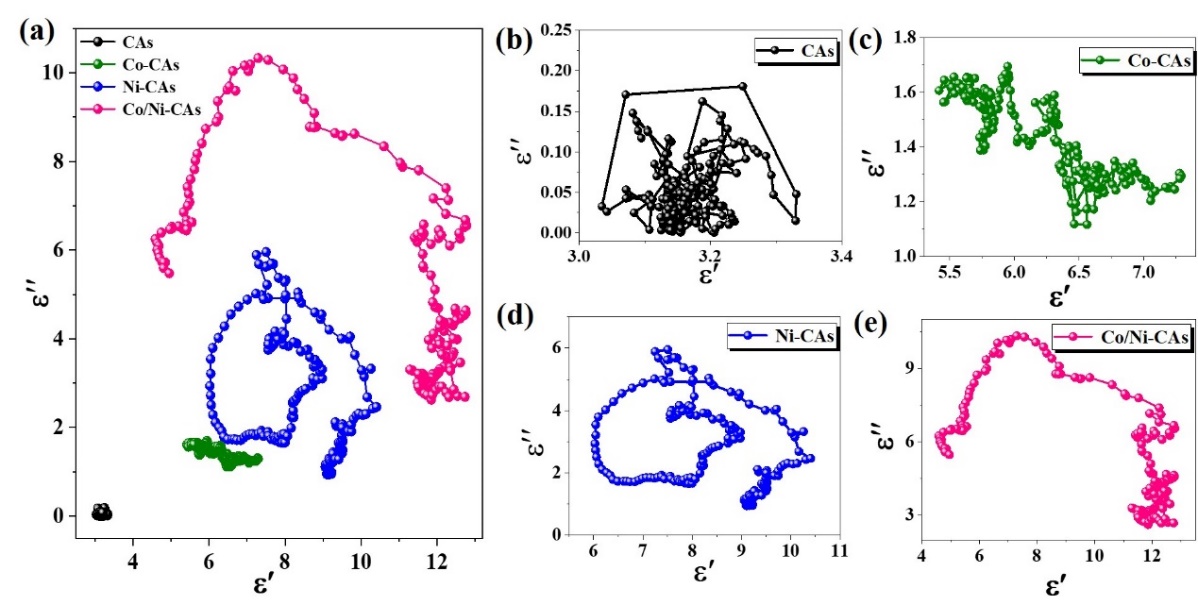
**

**Fig. S7** (**a**) *Cole-Cole* semicircles of samples. (**b**) CAs, (**c**) Co-CAs, (**d**) Ni-CAs and (**e**) Co/Ni-CAs


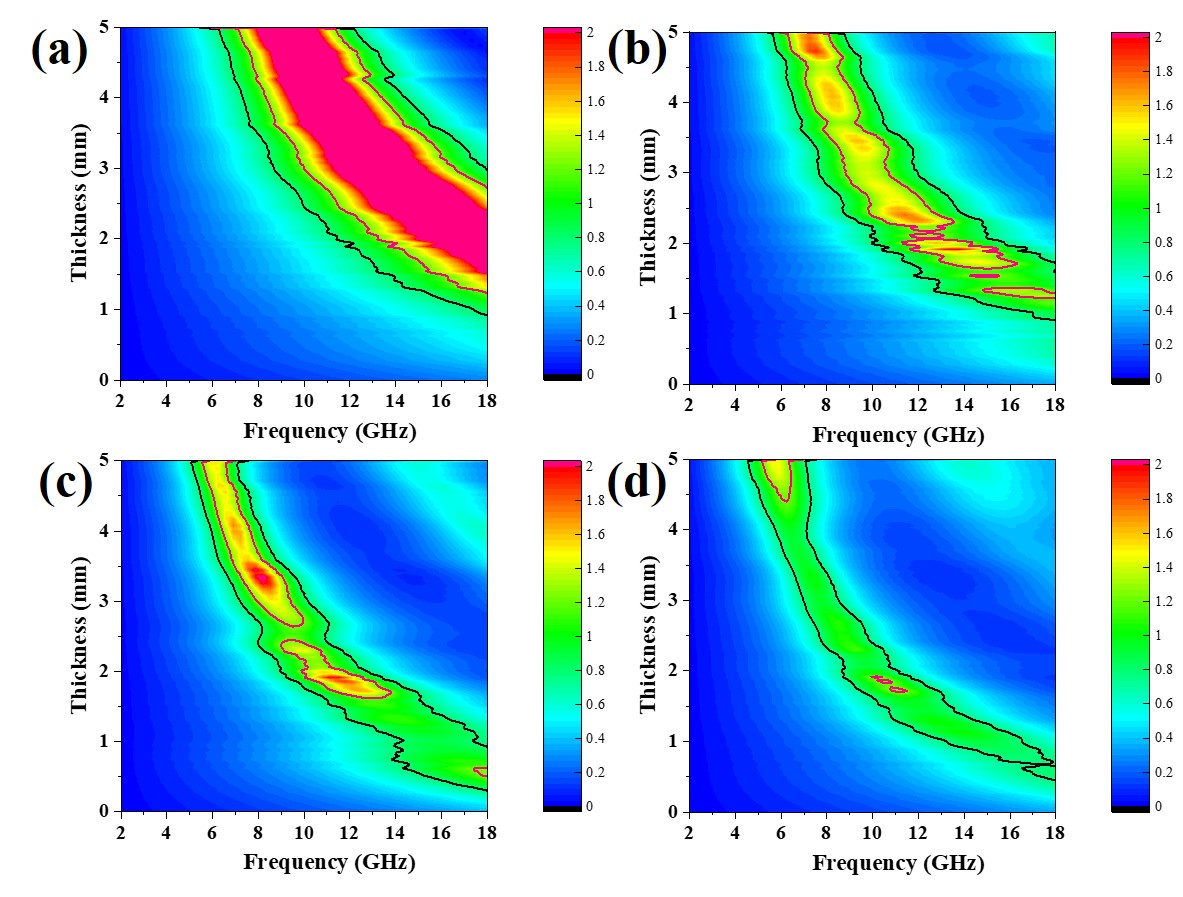


**Fig. S8** (**a-d**) 2D plots of impedance matching degree for samples

Generally, the normalized impedance Z can measure materials’ impedance matching level, and it is appraised by following equations according to the Maxwell′s equations:

$\text{Z = |}\text{Z}_{\text{in }}\text{/}\text{Z}_{\text{0}}\text{|=}\sqrt{\frac{\mu_{r}}{\text{ε}_{\text{r}}}}\text{tanh}\left( \text{j}\frac{\text{2πfd}}{\text{c}}\sqrt{\mu_{r}\text{ε}_{\text{r}}}\text{ } \right)$

where Z_0_ and Z_in_ embody the input impedance of the air and absorber, *d* is the thickness of absorber, *f* is the frequency of an electromagnetic wave and *c* represent the velocity of light in a vacuum.

The larger area between Z= 0.8 (black solid lines) and Z= 1.2 (red solid lines), the better impedance matching characteristic of absorber.

**
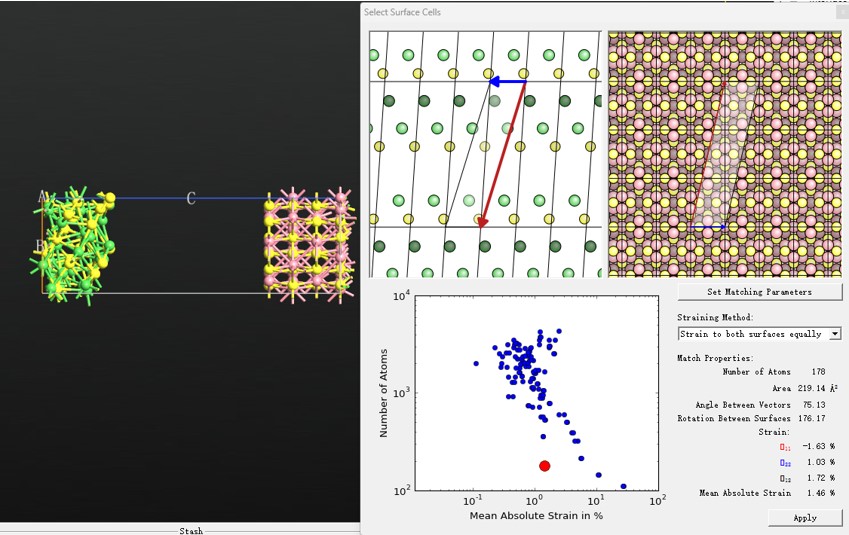
**

**Fig. S9** Constructing Co_9_S_8_(220)/Ni_9_S_8_(114) heterointerface model by Quantum ATK software


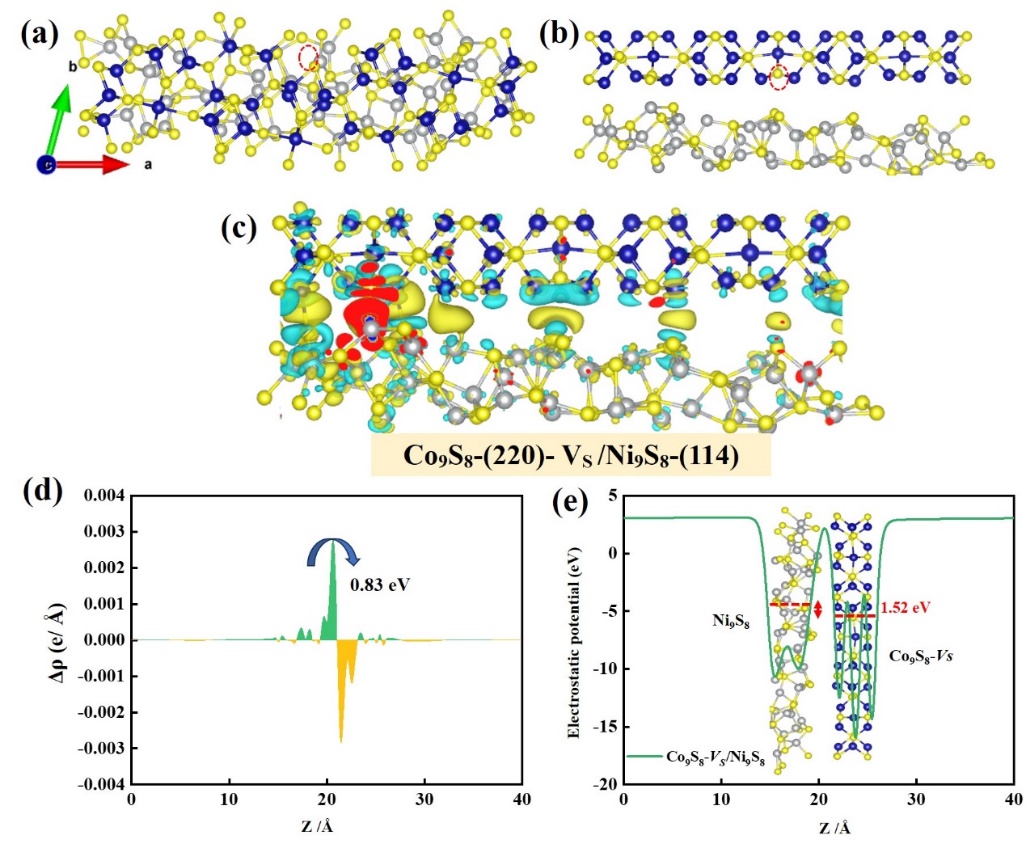


**Fig. S10** (**a**) Front and (**b**) top view, (**c**) charge density difference as well as (**d**) the planar-averaged charge density difference along the Z direction *Δρ*(z) and (**e**) electrostatic potential of Co_9_S_8_-*Vs*/Ni_9_S_8_ models

**Table S1** Phase composition of M-CAs

| Samples | CAs | Co-CAs | Ni-CAs | Co/Ni-CAs |
| --- | --- | --- | --- | --- |
| Products | CaS | CaS  Co_9_S_8_ | CaS  Ni_9_S_8_ | CaS  Ni_9_S_8_  Co_9_S_8_ |

**Table S2** Binding energies (eV) of all samples and the shift values of binding energy of Co/Ni-CAs, Ni-CAs and Co-CAs relative to CAs

|  | Binding energy of samples | | |  | Shift values of binding energy  relative to F-0 | | | |  |
| --- | --- | --- | --- | --- | --- | --- | --- | --- | --- |
| **Samples** | **S 2p_3/2_**  (eV) | **Co**  **2p_3/2_**  (eV) | **Ni 2p_3/2_**  (eV) | **Ca 2p_3/2_**  (eV) | | Δ**S2p_3/2_**  (eV) | Δ**Co 2p_3/2_**  (eV) | Δ**Ni 2p_3/2_**  (eV) | Δ**Ca 2p_3/2_** |
| **CAs** | 162.43 | -- | -- | 347.19 | 0 | | -- | -- | 0 |
| **Co-CAs** | 162.26 | 780.23 | -- | 347.32 | -0.17 | | 0 | -- | 0.13 |
| **Ni-CAs** | 161.62 | -- | 854.56 | 347.60 | -0.81 | | -- | 0 | 0.41 |
| **Co/Ni-CAs** | 161.48 | 781.13 | 855.26 | 347.73 | -0.95 | | 0.90 | 0.70 | 0.54 |

**Table S3** Binding energies (eV) of S 2p and the calculated sulfur vacancy concentration, -C-S=C bond for samples

|  |  | |  | | **S 2p** | |  |  | |
| --- | --- | --- | --- | --- | --- | --- | --- | --- | --- |
|  | 163.3(eV) | 162.5(eV) | | 161.4 (eV) | |  | | |  |
| **Samples** | **-C-S=C-** | **S2p_1/2_** | | **S2p_3/2_** | | **S2p_1/2_/Total (%)** | | | **-C-S=C/Total (%)** |
| **CAs** | 299.6 | 559.3 | | 987.3 | | 30.3 | | | 16.2 |
| **Co-CAs** | 95.6 | 163.6 | | 252.7 | | 32.0 | | | 18.7 |
| **Ni-CAs** | 84.2 | 156.3 | | 154.5 | | 39.6 | | | 21.4 |
| **Co/Ni-CAs** | 196.5 | 231.1 | | 137.9 | | 40.9 | | | 34.8 |

**Table S4** Comparison of EMW absorption performance of some representative sulfides-based absorbers

| **Absorber** | | ***RL*_min_ (dB)** | **EAB (GHz)** | **Matching**  **Thickness *d* (mm)** | **Refs.** |
| --- | --- | --- | --- | --- | --- |
| Ni_x_S_y_/Co_x_S_y_@C | -47.20 | | 3.70 | 2.50 | [S1] |
| WS_2_/NiO | -53.31 | | 4.88 | 2.22 | [S2] |
| ZnO/ZnS/CuS | -43.88 | | 5.10 | 1.59 | [S3] |
| CuS/MXene | -45.30 | | 5.20 | 2.00 | [S4] |
| NiS_2_@MoS_2_ | -18.13 | | 5.17 | 1.60 | [S5] |
| CuCo_2_S_4_/C | -52.60 | | 6.00 | 2.00 | [S6] |
| Cu_2_S/Cu_31_S_16_ | -15.10 | | 6.20 | 2.30 | [S7] |
| CoS_2_@MoS_2_/rGO | -58.00 | | 6.24 | 2.40 | [S8] |
| CaS/Ni_9_S_8_/Co_9_S_8_@C | -48.30 | | 6.76 | 1.80 | **This work** |

**Table S5** Electrostatic potential of samples

| **Samples** | Ni_9_S_8_ | Co_9_S_8_ | electrostatic potential difference (eV) |
| --- | --- | --- | --- |
| Co_9_S_8_/Ni_9_S_8_ | -8.01 | -8.34 | 0.33 |
| Co_9_S_8_/Ni_9_S_8_-*Vs* | -7.52 | -9.65 | 2.13 |
| Co_9_S_8_-*Vs*/Ni_9_S_8_ | -7.998 | -9.52 | 1.52 |

**Supplementary References**

1. L. Gai, G. Song, Y. Li, W. Niu, L. Qin et al., Versatile bimetal sulfides nanoparticles-embedded N-doped hierarchical carbonaceous aerogels (N-Ni_x_Sy/CoxSy@C) for excellent supercapacitors and microwave absorption. Carbon **179**, 111–124 (2021). <https://doi.org/10.1016/j.carbon.2021.04.029>
2. D. Zhang, Y. Xiong, J. Cheng, J. Chai, T. Liu, X. Ba, S. Ullah, G. Zheng, M. Yan, M. Cao, et al., Synergetic dielectric loss and magnetic loss towards superior microwave absorption through hybridization of few-layer WS_2_ nanosheets with NiO nanoparticles. Sci. Bulletin **65,** 138-146 (2020). <https://doi.org/10.1016/j.scib.2019.10.011>
3. G. Chen, L. Zhang, B. Luo, H. Wu, Optimal control of the compositions, interfaces, and defects of hollow sulfide for electromagnetic wave absorption. J. Colloid Interface Sci. **607**, 24–33 (2022). <https://doi.org/10.1016/j.jcis.2021.08.186>
4. G. Cui, L. Wang, L. Li, W. Xie, G. Gu, Synthesis of CuS nanoparticles decorated Ti_3_C_2_T_x_ MXene with enhanced microwave absorption performance. Prog. Nat. Sci. Mater. Int. **30**, 343–351 (2020). <https://doi.org/10.1016/j.pnsc.2020.06.001>
5. X.-J. Zhang, S.-W. Wang, G.-S. Wang, Z. Li, A.-P. Guo et al., Facile synthesis of NiS_2_@MoS_2_ core-shell nanospheres for effective enhancement in microwave absorption, RSC Adv. **7,** 22454-22460 (2017). <https://doi.org/10.1039/C7RA03260A>
6. Q. Ma, Z. Xu, X. Li, X. Cheng, Core-shell CuCo_2_S_4_ based microspheres composited with carbon black nanoparticles for effective microwave absorption, J. Alloys Compd. **938**, 168577 (2023). <https://doi.org/10.1016/j.jallcom.2022.168577>
7. W. Li, J. Chen, P. Gao, MOFs-derived hollow Copper-based sulfides for optimized electromagnetic behaviors. J. Colloid Interface Sci. **606**, 719–727 (2022). <https://doi.org/10.1016/j.jcis.2021.08.019>
8. T. Zhu, W. Shen, X. Wang, Y.-F. Song, W. Wang, Paramagnetic CoS**_2_**@MoS**_2_** core-shell composites coated by reduced graphene oxide as broadband and tunable high-performance microwave absorbers. Chem. Eng. J. **378,** 122159 (2019). <https://doi.org/10.1016/j.cej.2019.122159>
